# Enhancement of strength and toughness of bio-nanocomposites with good transparency and heat resistance by reactive processing

**DOI:** 10.1016/j.isci.2022.104560

**Published:** 2022-06-08

**Authors:** Hengti Wang, Chenyan Rong, Jichun You, Yongjin Li

**Affiliations:** 1 College of Material, Chemistry and Chemical Engineering, Key Laboratory of Organosilicon Chemistry and Material Technology, Ministry of Education, Hangzhou Normal University, Hangzhou, Zhejiang 311121, People’s Republic of China

**Keywords:** Materials science, Materials class, Biomaterials

## Abstract

Growing concerns in addressing environmental challenges are driving the rapid advancement of both bio-based and environmental friendly materials. Biodegradable polymers have been compounded with various nanofillers to fulfill the multiple requirements in real applications. However, current technologies remain to be improved in terms of the intrinsic inferior performance and the lack of interfacial interactions. In this work, we employed a facile route to develop bio-nanocomposites integrating multiple functionalities by reactive processing of polylactide and reactive boehmite nanorods. The grafting of polymer chains onto the surface of the nanorods encourages fully homogeneous dispersion of nanofillers with even 30 wt% loadings. Such nanocomposites exhibit simultaneously enhanced tensile strength, modulus, ductility, and impact strength. Moreover, the bio-based nanocomposites present promising features such as high transparency, improved flame resistance, and heat resistance. This work demonstrates exciting opportunities to produce bio-plastics with diverse functionalities in versatile applications of sustainable packaging industry and engineering plastics.

## Highlights

• Bio-nanocomposites integrating multiple functionalities are fabricated
• Fully homogeneous dispersion of boehmite nanorods is achieved
• Homogeneous dispersed nanorods induced forced high elastic deformation of polylactic acid (PLLA) matrix

## Graphical abstract

**Figure:**
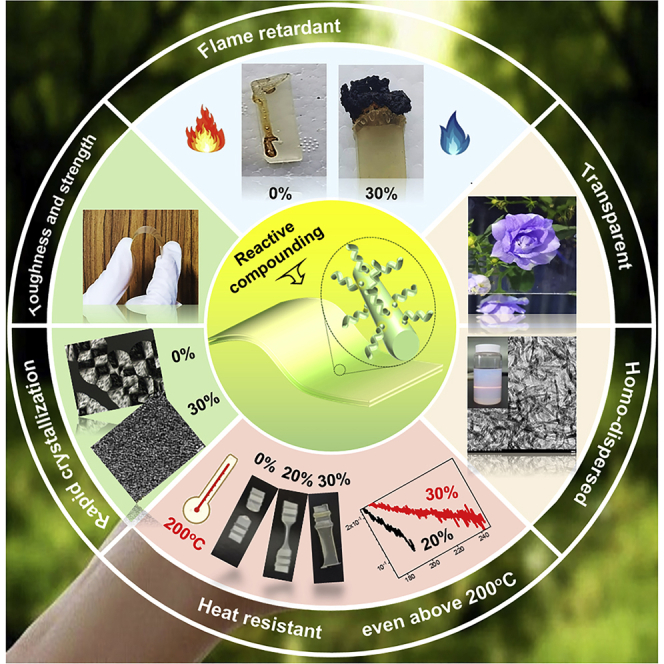

## Introduction

Plastics, mainly derived from fossil resources (Weckhuysen, 2020; Zhao et al., 2022), are ubiquitous for human beings, ranging from daily necessities (e.g., packaging (Pernot et al., 2002; Shi et al., 2022), textile (Mu and Yang, 2022), and construction (Zaidy et al., 2019)), to advanced engineering fields (e.g., aircraft (Borba et al., 2020), automobile (Gao et al., 2018; Toledo et al., 2011), and electron (Boutry et al., 2018; Vieira et al., 2022) industry). Nevertheless, high durability has made the annually accumulated petrochemical-based materials one of the major global challenges (Jordan et al., 2018; Kakadellis and Rosetto, 2021; Xia et al., 2021). Reports (Brahney et al., 2020; Geyer et al., 2017; MacLeod et al., 2021) estimated that approximately 11 billion metric tons of plastic waste will be accumulated by 2025, seriously contaminating parts of the world (Law et al., 2020; Rochman et al., 2016). There is an urgent need to exploit sustainable replacement to obviate the plastic threat. Therefore, biodegradable polymers have attracted significant attention as the promising alternatives to conventional non-degradable counterparts (Altman et al., 2021; Jiang et al., 2020; Sharma et al., 2022; Zhu et al., 2016). So far, the commercially available biodegradable polymers are far from the high requirements for real applications, especially in the engineering fields, owing to the poor physical properties of the inherent molecular chain structure. Nanofillers are therefore incorporated into the biodegradable polymer matrix to achieve enhanced physical properties (Li et al., 2012; Mohanty et al., 2018). However, current technologies for nanocomposites are still confronted with major challenges that constrain large manufacturing. Firstly, it remains great challenging to design high-performance sustainable materials with exceptional toughness and mechanical strength. Generally, inorganic nanofillers can improve the modulus and tensile strength, but will lead to a significant decrease in toughness (Song and Wang, 2020; Zhang et al., 2019). Yu et al. (2005) established a pioneering work on nylon 6/montmorillonite nanocomposites with synchronously improved stiffness, strength, and toughness with the aid of water. Unfortunately, the exceptions are still rare, especially by using traditional processing equipment. Secondly, nanofillers and bio-matrix are intrinsically immiscible and aggregation of nanofillers is inevitably occurred especially at high content (Ding et al., 2021; Jiang et al., 2020; Roy Goswami et al., 2021; Wang et al., 2021), resulting in the compromising of multi-functionalities toward versatile applications. For example, it is critically difficult to achieve transparent nanocomposites owing to the aggregation of the fillers in the transparent polymer matrix (Liu et al., 2021; Sato et al., 2020). High transparency is usually, indeed, important for applications in the packaging industry. Thirdly, toxic solvents associated with complex fabrication steps are often required especially in academia, posing severe issues regarding eco-friendly and cost-efficiency (Wang et al., 2020, 2022).

**Figure 1:**
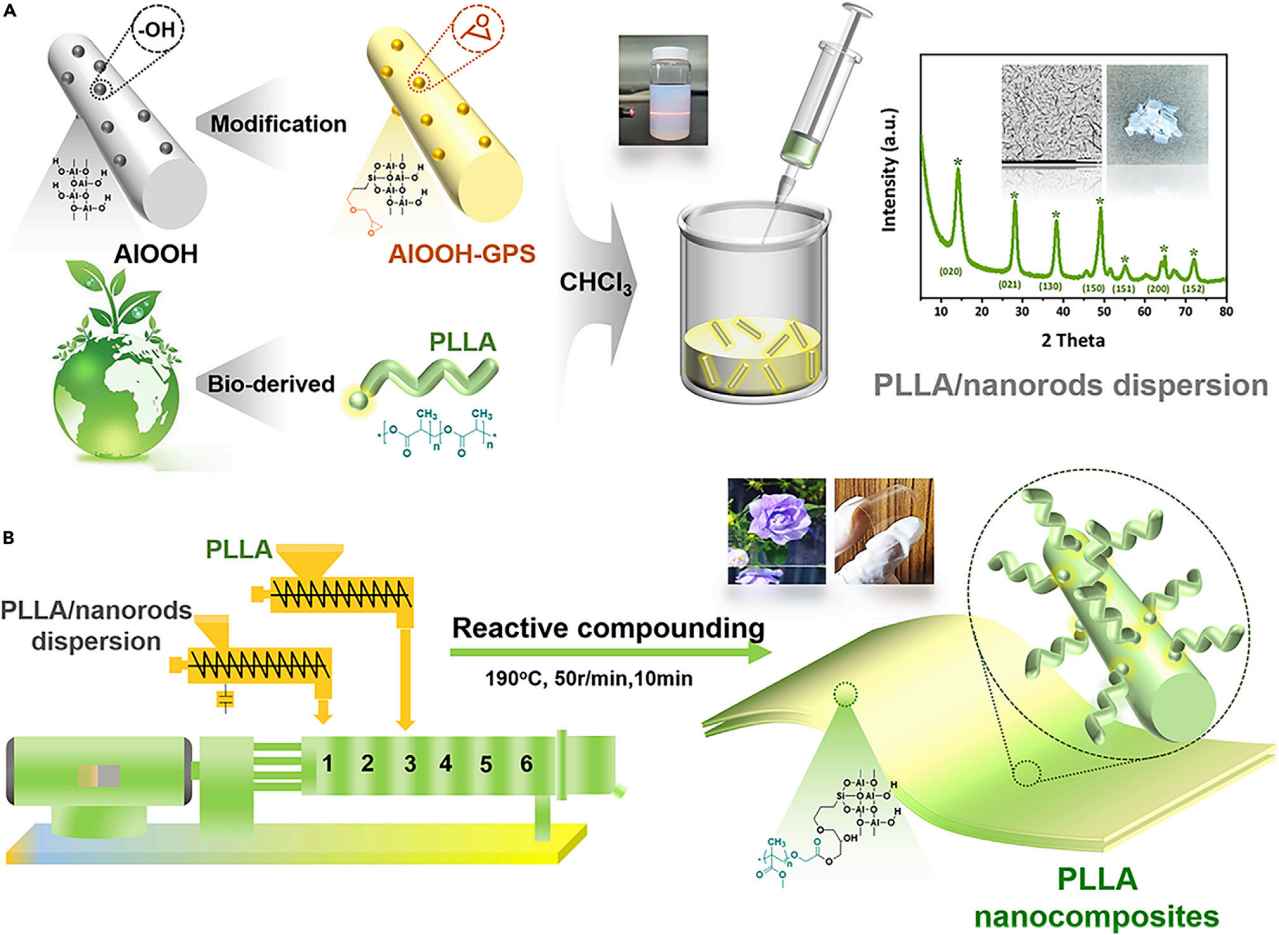
Reactive compounding enabled PLLA nanocomposites (A and B) (A) Schematic diagram of the fabrication strategy of PLLA/nanorods’ dispersion, (B) PLLA nanocomposites through reactive compounding process.

To address the above critical problems, we developed an industrially relevant strategy to fabricate advanced polylactic acid nanocomposites integrating superior mechanical property, multiple functionalities (Figure 1). Polylactic acid (PLLA) (Hamad et al., 2018; Li et al., 2021) is utilized as a biodegradable matrix because it is predominantly utilized in the rational design of renewable nanocomposites with promising features such as facile processability (Zhao et al., 2020), mechanical stiffness (Jin et al., 2019; Wu et al., 2021), and biocompatibility (Chen et al., 2021; Muller et al., 2017). Surface-modified boehmite nanorods (AlOOH-GPS termed as AG) containing reactive epoxide groups were pre-made according to our previous work (Fu et al., 2019; Hu et al., 2021; Li et al., 2020; Rong et al., 2022) (Figure 1A). Then, PLLA and AG were mixed and reacted in a batch mixer (Figure 1B). Note that the design of bio-nanocomposites involves the processing equipment, in which the dispersion of nano-fillers in a polymer matrix remains a key and yet poorly understood issue (Hu et al., 2007). *In-situ* grafting reaction between terminal carboxylic groups of PLLA and epoxide groups on AG nanorods occurred immediately (one to a few minutes) during processing. The detailed kinetics of the reaction between epoxy and carboxylic acid in heterogeneous polymer melts has been verified previously (Hu et al., 1997). Therefore, a fully degradable polymer matrix is deconstructed to display uniformly nanorods dispersed composites with sufficient physical entanglements between the inorganic and organic interfaces. Unlike most strategies, the bio-nanocomposites can be homo-dispersed at a single rod level even at very high nanofiller loadings (30 wt%), resulting in the unexceptional combination of the two components synergistically. (i) The bio-plastic is mechanically strengthened and toughened, manifesting an abnormal rigid-toughness balance, which neither traditional plastic nor nanocomposites can achieve. (ii) Multiple functionalization incorporating superior transparency, flame resistance, and heat resistance is simultaneously integrated. The effects of AG on the morphology, mechanical performance, optical properties, thermal properties, and combustion performance of such nanocomposites were systematically investigated. The strong, tough, transparent, thermo-stable, and biodegradable alternatives via industrially relevant methods are satisfactory to fulfill multiple requirements regarding the enormous usage of plastic replacement, especially in sustainable packaging and engineering plastics.

## Results

### Dispersion of nanorods in the polylactic acid matrix

**Figure 2:**
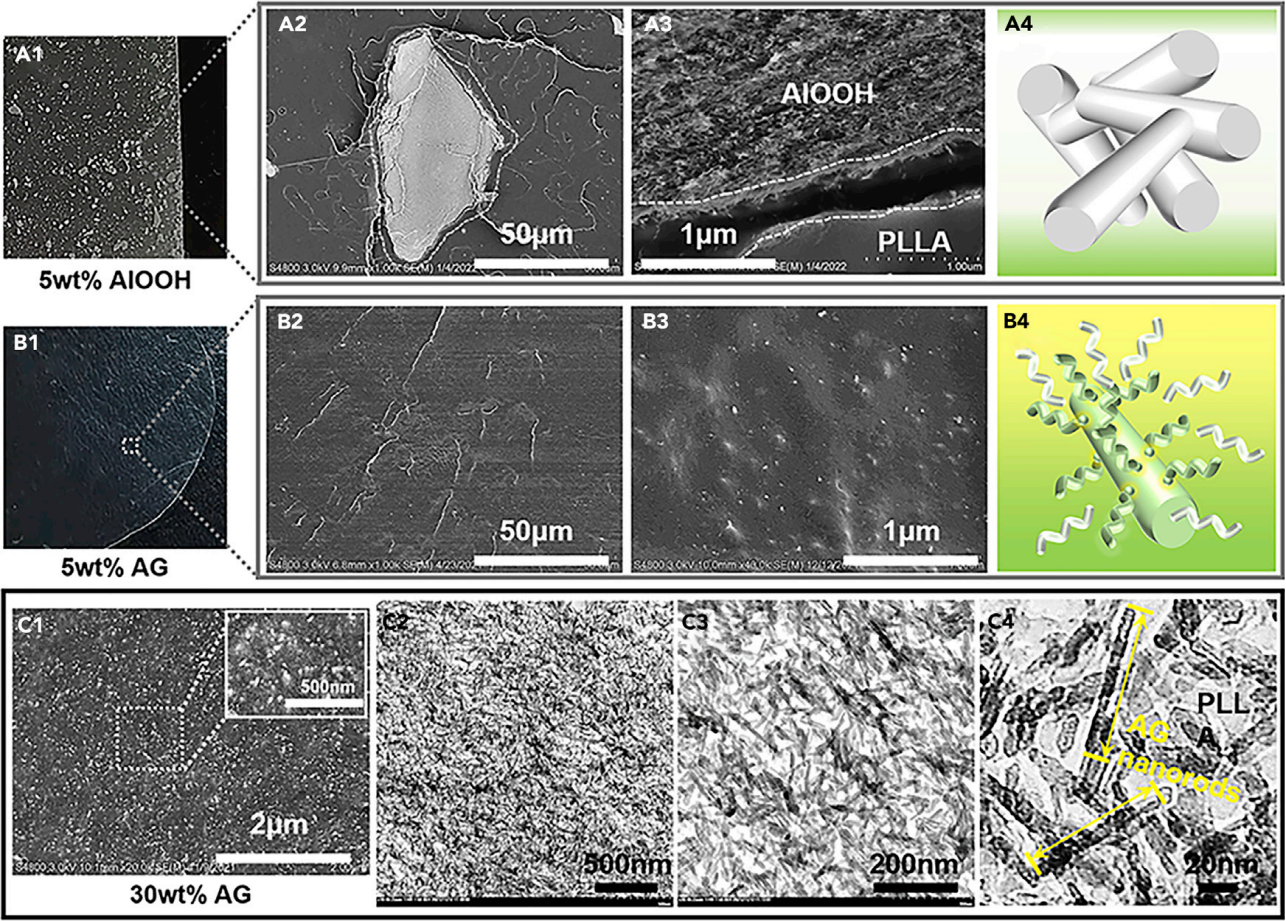
Homogeneous dispersion of AG nanorods (A–C) Photographs and corresponding SEM, TEM images of the PLLA nanocomposites containing (A) 5 wt % AlOOH nanorods, (B) 5 wt % and (C) 30 wt % AG nanorods.

It is a great challenge for the perfect dispersion of nanofillers in polymer matrix owing to the inherent immiscibility. In this work, boehmite nanorods have been synthesized and modified carefully according to previous work (Hu et al., 2021; Li et al., 2020; Rong et al., 2022) (Equation S1). The nanorods have a diameter of about 5 nm and a length of 100 nm (Figure S1). The epoxide groups have been grafted onto the surface of the nanorods (Figure S2) by the silane coupling agent so that the expected reaction between the carboxylic acid groups of PLLA with the epoxide groups occurs during the melt processing (Figures S3 and S4). It is seen that the pristine AlOOH nanorods without surface modification aggregate into the large agglomerations of about 30 μm in the PLLA matrix at the loading of 5 wt % (Figure 2A_1-3_). The pristine nanorods are hydrophilic and they are totally immiscible with the hydrophobic PLLA matrix. Moreover, the nanosize of the filler leads to strong interactions between the nanorods (Figure 2A_4_). The shear stress during the melt blending cannot overwhelm such interactions (Hu et al., 2007) and the large nanorods agglomerates were observed with even 5 wt % loadings. A significant different situation was found for the surface-modified nanorods. Perfect dispersion without any aggregation was observed for the nanocomposites with the epoxide group modified nanorods. One can see the homogeneous dispersion of the single AG nanorod in the PLLA matrix over the different magnifications (Figure 2B_1-3_). Such perfect dispersion can be attributed to the two reasons. On the one hand, the surface modification improves the miscibility between the nanorods and the matrix. More important, the reaction between the carboxylic acid groups of PLLA with the epoxide groups leads to the grafting of PLLA long chains onto the surface of the nanorods. The calculated grafting ratio of PLLA on AG nanorods is as high as 41.7 wt% (Figure S4). The grafted PLLA chains entangle with the free PLLA chains and the single nanorod can be well dispersed in the polymer matrix (Figure 2B_4_). The covalent bonding of the PLLA chains at the end with the nanorods plays a critical role to nucleate PLLA matrix (Figure S5). Moreover, it encourages the simultaneous increase in the modulus (tensile strength) and the toughness (see the following section). The fact that surface-modified nanorods are homogeneously dispersed in the PLLA matrix by the reactive compounding is not dependent on the filler loadings. As shown in Figure 2C, perfect homogeneous dispersion can also be achieved with even 30 wt % nanorods’ loadings in the nanocomposites. No any aggregations is found in both SEM and TEM images (detailed SEM observation of nanocomposites with various contents of AG nanorods is shown in Figure S6). Moreover, all the nanorods keep the original shape and size after the reactive compounding from the TEM images (Figure 2C_4_). Such perfect dispersion and the nanosize of the fillers give the perquisite of the transparency of the final nanocomposites.

### Mechanical performance

**Figure 3:**
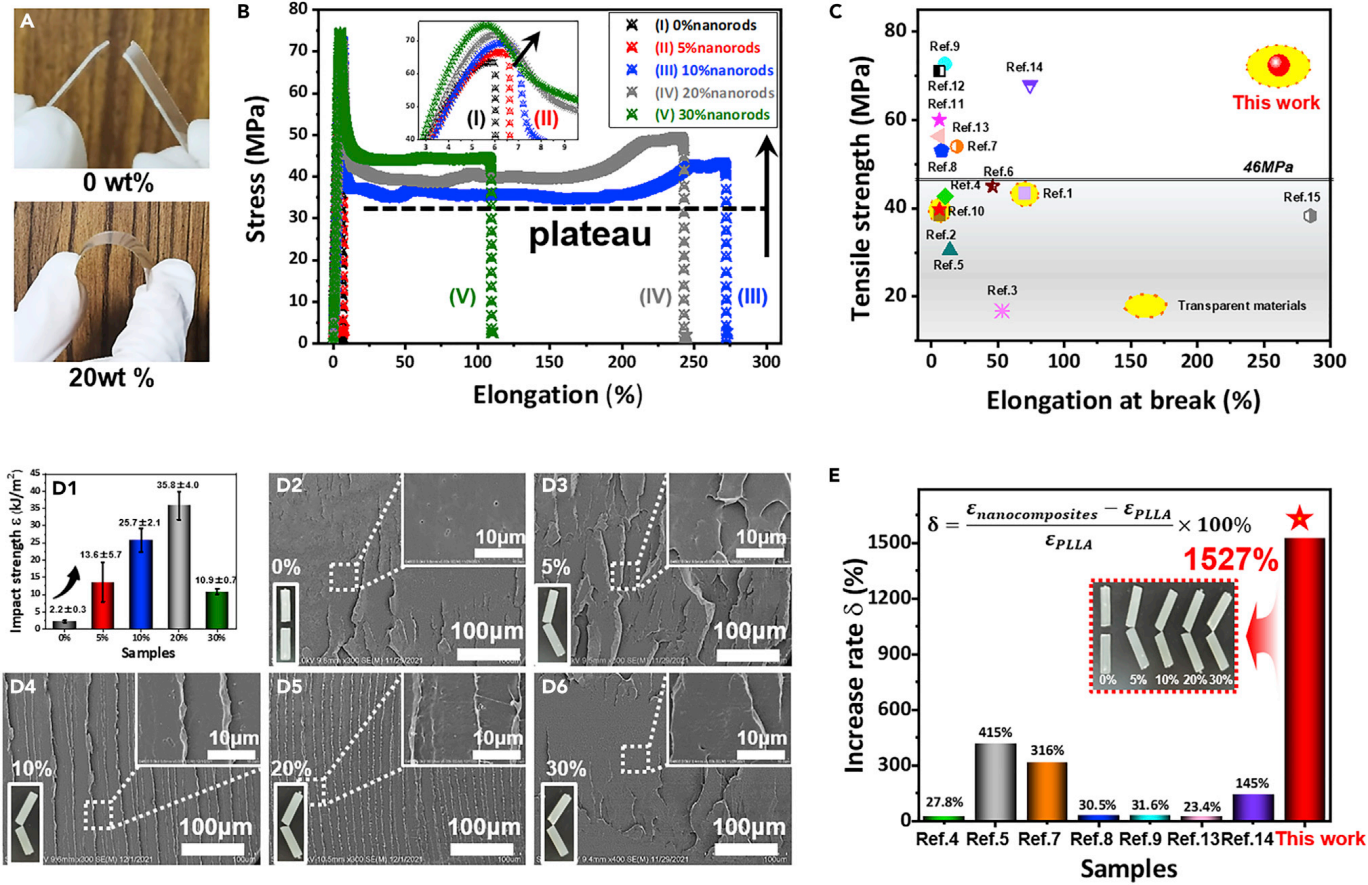
Improved mechanical performance (A–E) (A) Photographs of PLLA and the nanocomposites containing 20 wt % nanorods, (B) Representative stress-strain curves, (C) Significant improvement for comparing the mechanical performance of reported PLLA nanocomposites, (D1) Impact strength of PLLA nanocomposites with various concentrations of nanorods, and SEM images of impact sections of PLLA nanocomposites with (D2) 0 wt%, (D3) 5 wt%, (D4) 10 wt%, (D5) 20 wt% and (D6) 30 wt% of modified nanorods, (E) Increase rate (δ) of impact strength comparing with reported PLLA nanocomposites (in the inserted equation, εnanocomposites and *ε*_*PLLA*_ referred to impact strength or impact energy of PLLA-based nanocomposites and pristine PLLA, respectively). Details are given in Tables S2–S4.

The mechanical properties, such as Young’s modulus, tensile strength, ductility, and toughness, are critically important for the real applications of polymer materials. So far, either strengthening or toughening can be achieved by compounding of inorganic fillers or elastomers into the polymer matrix (Song and Wang, 2020; Yu et al., 2005; Zhang et al., 2019). The simultaneous enhancement in both tensile strength (modulus) and the impact strength (including ductility and toughness) has rarely been reported for the simple binary polymer blends or polymer nanocomposites. Here, we found that the homogeneously dispersed boehmite nanorods increased both strength and toughness of PLLA at the same time. Both neat PLLA and PLLA composites with non-modified pristine AlOOH nanorods are very brittle (Figure S7), but the PLLA nanocomposites with surface-modified nanorods are both strong and tough (Figure 3A). Figure 3B shows the stress-strain curve of PLLA nanocomposites with different AG nanorods (shortened as nanorods later) loadings. It is found that neat PLLA are rigid and brittle with the elongation at break and tensile strength of 5.9% and 67.0 MPa, respectively. 5 wt % nanorods leads to the increase in both elongation at break and tensile strength. The values increase to 6.5% and 69.5 MPa, respectively (Table S2). This means that a small amount of the modified nanorods improves the mechanical performance slightly. With the further increase of nanorods’ loadings, the nanocomposites show totally different tensile behaviors from the typical ductile stretching behaviors. The materials yield first followed by the yielding softening and the cold stretching, finally the strain hardening and break. The nanocomposites with 10 wt % nanorods have elongation at a break of 271.2% (Curve III), which is 46 times higher than that of neat PLLA. At the same time, the modulus and tensile strength of the material also gradually increased with increasing nanorods’ loadings, indicating both the strengthening effects and the improved ductility. In particular, the nanocomposites with 30 wt % nanorods (Curve V) showed the tensile yielding strength of about 75.1 MPa and the elongation at the break of 108.8%. Obviously, the inorganic nanorods strengthen the PLLA matrix drastically (SEM observation of tensile samples shown in Figure S8). By comparing the reported literature on the modification of PLLA, this work shows desirable tensile strength and ductility (Figure 3C). For instance, unexpected improvement in ductility (elongation at break ∼285%)) was achieved for the PLLA/MWCNTs-*g*-PLLA nanocomposites (*Ref. 15* shown in Table S4), accompanied by slightly compromise in tensile strength (38.3 MPa). This is because the PLLA chain grafted on nanorods can effectively physically entangle with the PLLA molecular chain in the matrix under the condition of high filling, and a good interface is formed between the uniformly dispersed nanorods and the PLLA matrix. Of note, tensile plateau as marked in Figure 3B drastically increased from 35 MPa (10 wt% nanorods), 41 MPa (20 wt% nanorods), to 44 MPa (30 wt% nanorods). The plateau value of 30 wt% incorporated nanocomposites is extremely high that exceeds the tensile strength reported in literature listed in Figure 3C. We believe that the maximized strengthening effect of nanofiller is achieved from its uniform dispersion and formation of nanorods network. Besides, almost all PLLA nanocomposites are not transparent in the reported literature. Here, the prepared PLLA nanocomposites with the surface-modified nanorods are transparent and they exhibit excellent mechanical properties compared with other reported PLLA composites, as marked by yellow circles in Figure 3C (Ref. 1: Erlantz et al., 2016; Ref. 2: Zhu et al., 2014; Ref. 3: Liang et al., 2019; Ref. 4: Huang et al., 2018; Ref. 5: Li et al., 2016a, ; Ref. 6: Jia et al., 2016; Ref. 7: Zhang et al., 2012; Ref. 8: Elsawy et al., 2016; Ref. 9: Zou et al., 2016; Ref. 10: Fallahi et al., 2017; Ref. 11: Wang et al., 2017; Ref. 12: , 2016b; Ref. 13: Gu et al., 2019; Ref. 14: Wen et al., 2015; Ref. 15: Amirian et al., 2013). The detailed optical properties will be discussed in the following section.

Toughness is important for many applications including packaging and injection molding parts. It is well known that neat PLLA is brittle with very low impact strength (Jin et al., 2019; Wu et al., 2021). The impact strength of the nanocomposites has been measured and plotted as a function of the nanorods’ loadings. It is very interesting to find that the impact strength of the material gradually increased with the increase of nanorods content followed by the decrease at very high loadings (Figure 3D_1_). The impact strength of 20 wt % nanorods sample is as high as 35.8 kJ/m^2^, which is about 15 times higher than that of neat PLLA. Even with the addition of 30 wt % nanorods, the toughness of the material is as high as 10.9 kJ/m^2^, 4 times higher than the neat PLLA.

The corresponding results can be observed in the SEM images of the impact section. The neat PLLA forms a fairly flat fracture surface, showing a typical brittle fracture (Figure 3D_2_). The nanocomposites with 20 wt % nanorods exhibit a relatively rough appearance and a considerable deformation of the matrix occurred during the impact test (Figure 3D_3-5_). This means that the PLLA with nanorods absorbs impact energy and induces the deformation. Moreover, we have also compared the toughness increase rate of this work with the reported literature and shown in Figure 3E. Highest increase rate (415%) was realized for the PLLA/*g*-CHW (Rod-like chitin whiskers) nanocomposites (*Ref. Five* shown in Table S4). To the best of our knowledge, this work demonstrated highest enhancement in the impact strength (1527%).

### Optical performance

**Figure 4:**
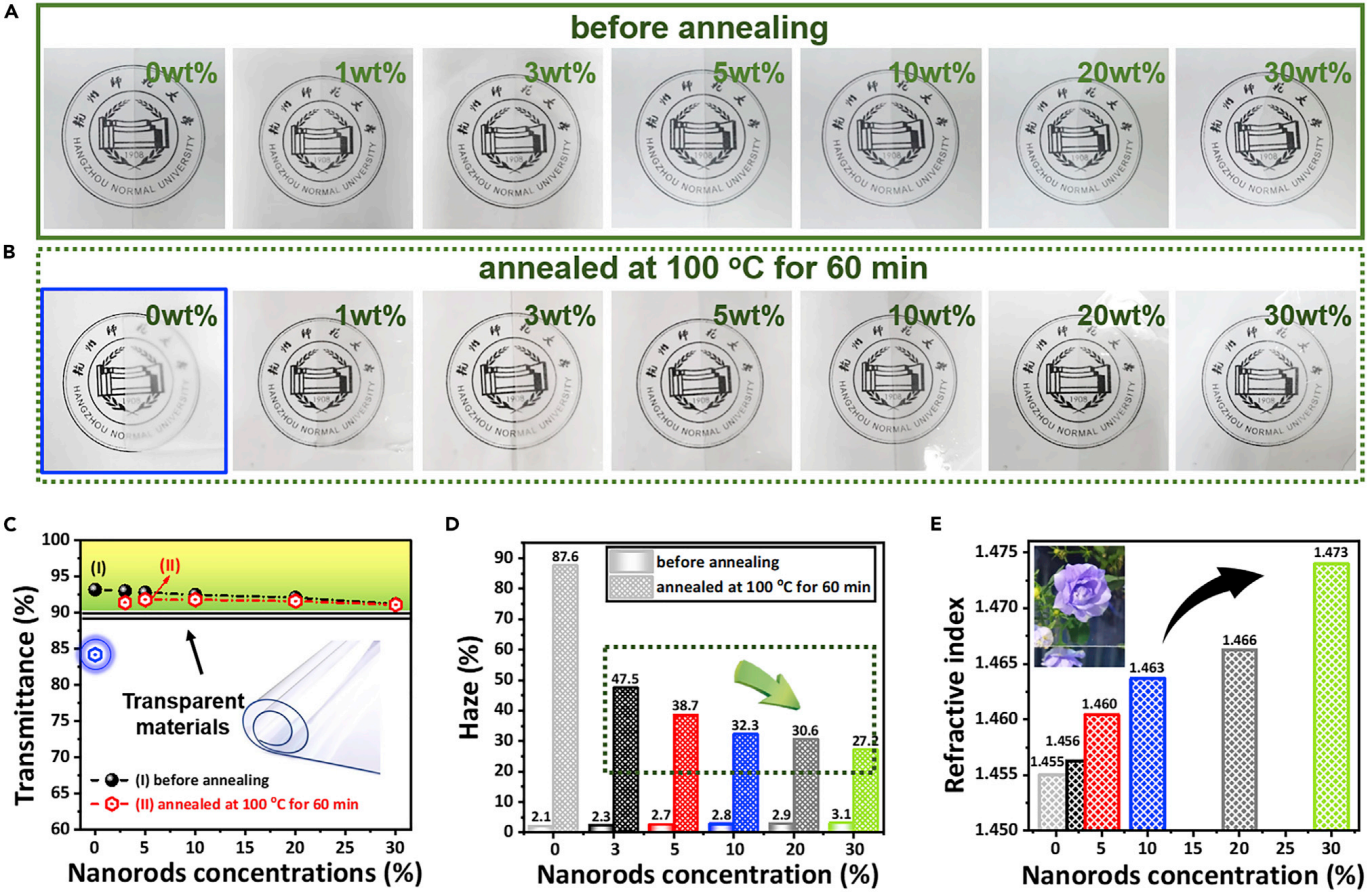
Improved optical performance (A–E) Photographs of PLLA nanocomposites containing various contents of nanorods (from 0%, 1%, 3%, 5%, 10%, 20%-30%, weight ratio) with a thickness of 100 μm: (A) before and (B) after annealing at 100°C for 60 min; (C) Light transmittance, and (D) Haze value of PLLA nanocomposites with different content of nanorods before and after annealing (E) Refractive index curves of PLLA nanocomposites with different content of nanorods.

The optical properties, including transmittance, haze, and refractive index, are important for various applications, especially for the packaging materials. It is well known that neat PLLA crystallizes slowly and the amorphous PLLA is highly transparent (Dong et al., 2012; Ye et al., 2015). However, the well-crystallized PLLA is opaque owing to the formation of large spherulites (Ye et al., 2015). The heterogeneity usually induces light reflection and refraction, which leads to low transmittance and high haze of materials. Moreover, most reported PLLA blends/nanocomposites are also not transparent because of the large domains and/or aggregates in the polymer matrix. We have investigated the nanorods’ loading effects on the transparency of PLLA before and after annealing at 100°C for 60 min (detail data are given in Supplementary Tables S5 and S6). The annealing induces the formation of the PLLA spherulites, which usually leads to dramatic increase in haze value. Figures 4A and 4B compared the photo of the PLLA nanocomposites with various amounts of nanorods before and after annealing. The thickness of the film keeps at a constant of 100 μm. It is also surprising that all the nanocomposites with even 30 wt % nanorods before annealing are highly transparent (the transmittance of 91.2% and the Haze value of 3.1%), very similar to the neat amorphous PLLA. This is obviously attributed to the perfect dispersion and the nanosize dimension of the nanorods. Any aggregates with a size larger than 100 nm will lead to a decreasing in the transmittance. The transmittance is as high as 91.1% for the nanocomposites with 30 wt % nanorods after annealing (Figure 4C). The annealing leads to a significantly increased Haze value because of the formation of large spherulites. As shown in Figure 4B, the PLLA film becomes opaque with a Haze value of 87.6% after the annealing, compared with the value of 2.1% before annealing. However, the homogeneously dispersed nanorods impede the growth of the spherulites and therefore decrease the Haze value even with the almost same crystallinity. High loading of nanorods presents the close distance between the neighboring nanorods and therefore induces the small spherulites, which gives low haze value and high transparency. One can find that the annealing leads to only a slight increase in the Haze value. As shown in Figure 4D, the haze value increases from 3.1% to 27% for the nanocomposites with 30 wt% nanorods after the annealing. This opens a new avenue to preparing transparent materials with both excellent optical properties and high heat resistance because one knows that the crystallization of PLLA definitely increases the heat distortion temperature.

Boehmite nanorods have higher refractive index of 1.631. It is interesting to investigate the refraction index of transparent nanocomposites. Refractive index is, indeed, a very important optical property for the optical lens. Figure 4E shows the refractive index of the PLLA/nanorods nanocomposites as the function of the nanorods’ loadings. Refraction is the ratio of the propagation speed of light in a vacuum to the propagation speed of light in a material. The refractive index of neat PLLA is 1.455. With the increase of nanorods content, the refractive index of the material gradually increases, and the refractive index of 30 wt% nanorods sample has the highest refractive index of 1.473 (Table S7).

### Thermal behaviors

**Figure 5:**
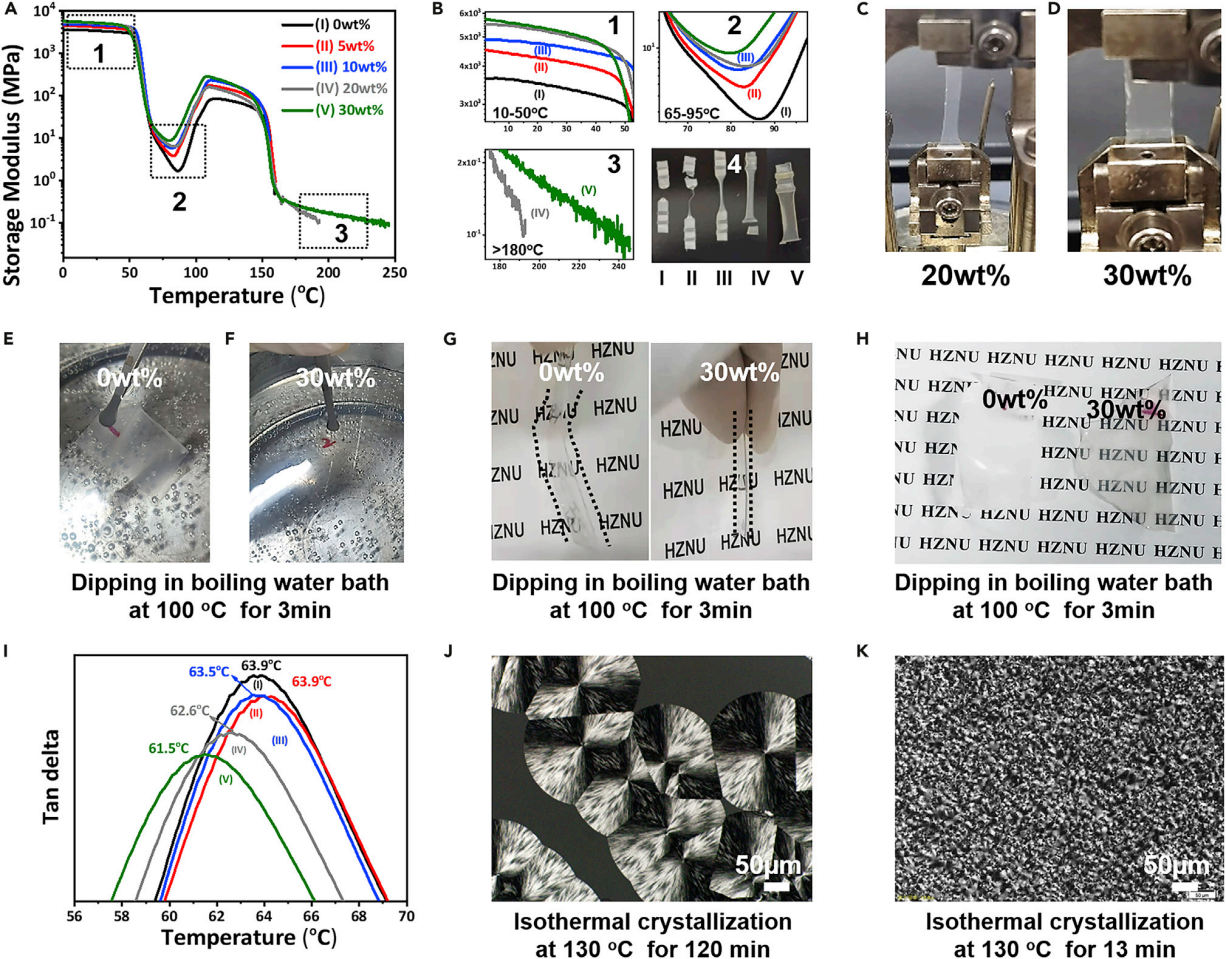
Improved thermal performance (A–K) Dynamic Mechanical Analysis (DMA): (A and B) storage modulus, (I) loss tangent values in dependence of temperature of PLLA nanocomposites; Heat resistance: (C and D) photographs of PLLA nanocomposites after DMA experiments, photographs of (E) PLLA and (F) PLLA nanocomposites with 30 wt % nanorods in boiling water bath (100°C) for 3 min; (G and H) photographs of PLLA and PLLA nanocomposites after dipping in boiling water for 3 min; Crystallization behaviors: POM images of isothermal crystallization of (J) pure PLLA and (K) PLLA nanocomposites with 30 wt% nanorods at 130°C.

Inorganic nanofillers usually improve the heat resistance of polymer materials (Hu et al., 2021; Li et al., 2020; Rong et al., 2022), especially for the homogeneously dispersed nanofillers at high loadings. The formation of inorganic nanofillers network in polymer matrix benefits the heat resistance. Figure 5A shows the DMA curves of PLLA/nanorods nanocomposites as a function of temperature for the quenched nanocomposites with the indicated nanorods’ loadings. The DMA measurements have also been carried out for the annealed samples (Figure S9, Table S8). All samples decrease drastically in the storage modulus over the temperature at about 60-70°C because of the glass transition of the PLLA matrix in the nanocomposites. The modulus was then recovered at the temperature of about 110-120°C owing to the cold crystallization of PLLA after the glass transition (Dong et al., 2012). Differences can be observed for the nanocomposites with various amounts of nanorods (detail given in Table S9). First, the storage modulus increases with increasing nanofiller loadings, indicating the strengthening effects of the inorganic nanofillers over the whole temperature range (Figure 5B_1_). Second, the starting temperature for the modulus enhancements decreases with increasing nanorods’ loadings (Figure 5B_2_). This means that the cold crystallization temperature of the PLLA matrix in the nanocomposites decreases with the addition of the nanorods, indicating the nucleation effects of well-dispersed nanorods. This is also beneficial to the injection cycling time for the nanocomposites and the optical properties of the small crystallites. Third, the nanocomposites with small amounts of nanorods (less than 20 wt %) melt at about 160°C and the samples were totally melted and broken (Figure 5B_4_). However, for the samples with 20 wt % (Figure 5C) and 30 wt % (Figure 5D) nanorods, the materials are still self-supported even at a temperature much higher than the melting temperature of the PLLA matrix. The nanocomposite with 20 wt % nanorods keeps the shape at 190°C while the sample with 30 wt% nanorods keeps the shape even at 240°C (Figure 5B_3_). This means that the heat resistance of PLLA is significantly enhanced by the nanorods. The nanorods form the scaffold (network) in the PLLA matrix at a high concentration.

It should be also noted that the boehmite nanorods increase the dimensional stability of the PLLA significantly. The neat PLLA thin-film shrinks and bends in hot boiling water (Figures 5E-5G). In contrast, no any shape changes were observed for the nanocomposites with 30 wt% nanorods (as compared in Figure 5G). Moreover, as indicated in the previous section, the boiling water treatment induces the clear film changing into the opaque film because of the formation of large spherulites. The nanocomposites keep a high clearity after immersing in the boiling water (Figure 5H).

The well-dispersed nanorods effects on the glass transition temperature (*T*_*g*_) of the PLLA matrix can be observed from the tan (delta) curves of the DMA analysis Figure 5I). It is very interesting to find that the *T*_*g*_ decreases with increasing nanorods’ loadings. The tan (delta) peak temperature (*T*_*g*_) of neat PLLA is 63.9°C and it gradually decreases to 61.5°C for the nanocomposites with 30 wt% nanorods (Table S8). Moreover, the relaxation peak of the PLLA matrix is also broadened with the incorporation of inorganic nanorods. The observed phenomenon indicates that the well-dispersed nanorods can efficiently increase the motions of PLLA chains. This is important for the physical property enhancement of the nanocomposites and we will discuss this in the later section. Similar results can also be found for the fully crystallized samples, as shown in Figure S9. The tan (delta) peak temperature (*T*_*g*_) of annealed PLLA is 69.1°C and it decreases to 62.4°C for the annealed nanocomposites with 30 wt% nanorods (Table S9). Such a big difference indicates the significantly changed PLLA chain relaxations with the addition of nanorods.

The nanorods’ effects on the melt crystallization behaviors of the PLLA matrix can be clearly observed from the isothermal crystallization. Neat PLLA crystallized very slow and crystallized into the very big spherulites with a diameter of about 200 μm after 120 min of crystallization (Figure 5J), so the fully crystallized PLLA films are opaque and the Haze value is high. In contrast, the homogeneously dispersed nanorods nucleate the PLLA and accelerate the crystallization of PLLA. The crystallization finished very quickly (13 min) and formed tiny crystals (Figure 5K). Detailed characterization on the crystal growth of PLA matrix with various AG loadings is given in Figures S10 and S11, Tables S10 and S11. It is indicated that AG nanorods can function as effective nucleating agents to accelerate the crystal growth of the PLA matrix. Accordingly, the nanocomposites keep high transparency and low Haze value even after the full crystallization of the PLLA matrix.

### Burning behaviors

**Figure 6:**
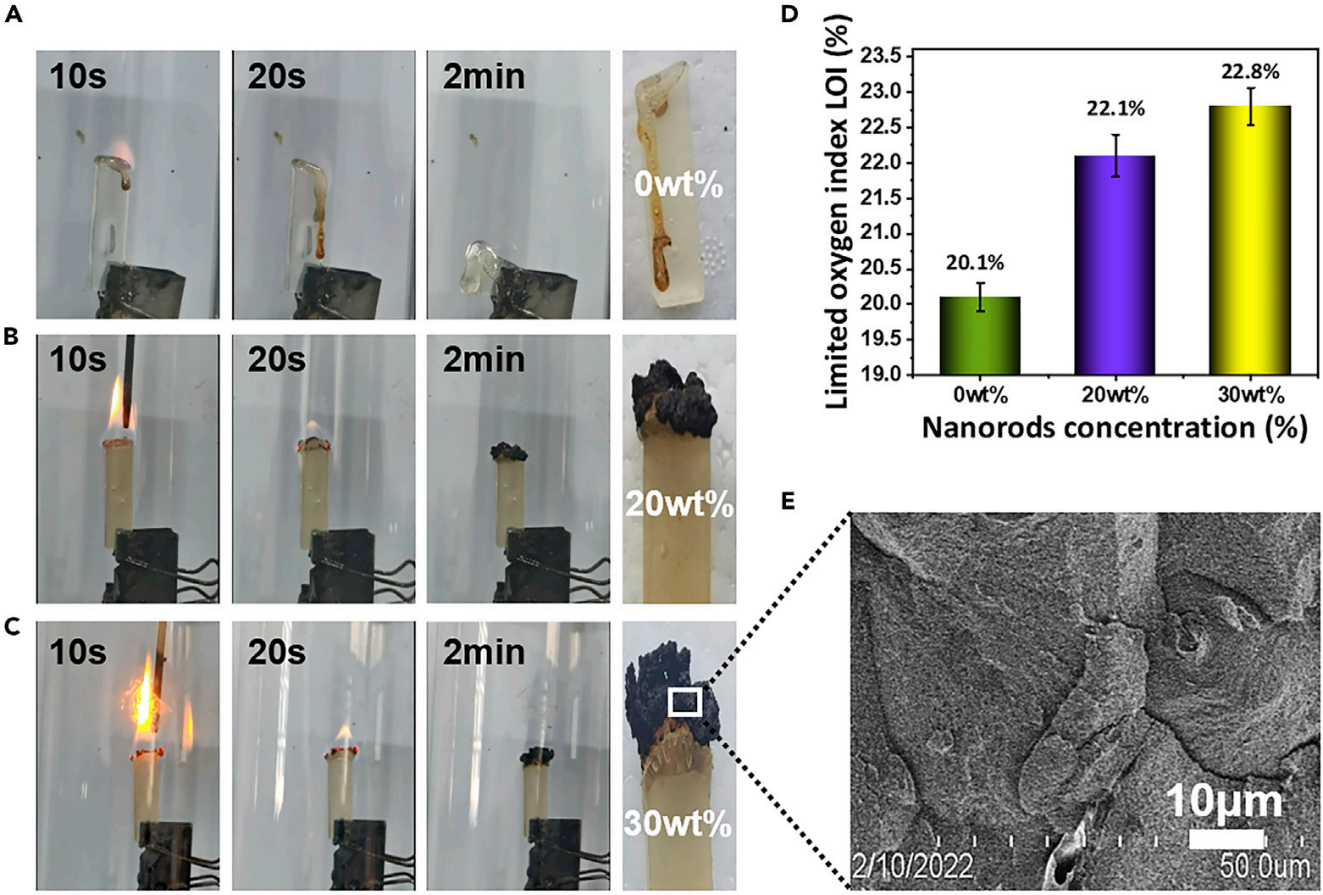
Improved flame resistance (A–E) Photographs of PLLA nanocomposites during combustion: (A) 0 wt%, (B) 20 wt%, and (C) 30 wt% nanorods, (D) LOI values of nanocomposites in dependence of nanorods’ loading, (E) SEM images of nanocomposites (30 wt%) after limiting oxygen index testing.

Boehmite is a kind of metal hydroxides and has been used as an effective flame retardant for polymers (Das et al., 2013; Li et al., 2020; Rong et al., 2022). PLLA itself is burnable with low LOI values and dropping during burning (Li et al., 2020). We expected that the homogeneously dispersed nanorods, especially the nanorods network in matrix, induce the flame retardancy of PLLA. It can be seen that neat PLLA is very flammable and the burning process is always accompanied by melting and dropping (Figure 6A, detailed limiting oxygen index measurements process of the samples are shown in Video S1). Remarkably, one can see clearly that the nanocomposites with 20 wt % (Figure 6B) and 30 wt % nanorods (Figure 6C) do not melt and drop during the burning. The two samples form a dense char layer in the burning fronts.

Video S1. Burning process of PLLA nanocomposites containing various contents of AlOOH nanorods from 0 wt %, 20 wt %, to 30 wt %, related to Figure 6D Burning process of bio-nanocomposites.

Obviously, the melting and dropping during the combustion of the PLLA can be greatly restrained by the incorporation of boehmite nanorods networks. The LOI value is increased from 20.1% (0 wt %), 22.1% (20 wt %), to 22.8% (30 wt %), indicating the increased flame resistance (Figure 6D). More important, we take a closer observation on the surface of nanocomposites’ sample after limiting oxygen index testing (Figure 6E). The surface of the nanocomposites formed complete carbon layers, which further indicates the flame retarding functions of the nanorods.

## Discussion

**Figure 7:**
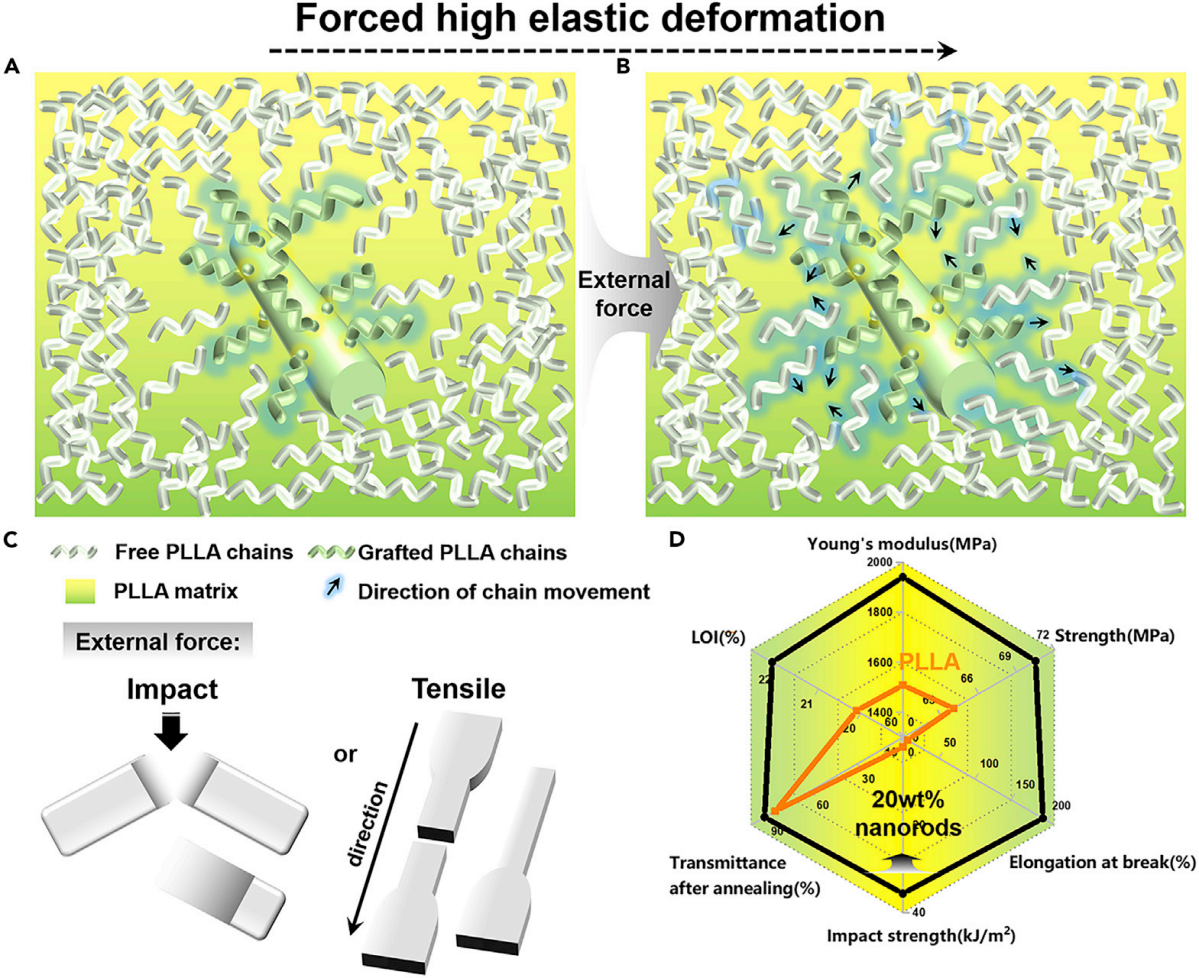
Schematic illustration of simultaneously enhanced tensile strength and impact strength (A–D) (A) Increased free volumes, (B), (C) Forced high elastic deformation of the nanocomposites by nanorods, (D) Radar plot showing the complex correlation among Young’s modulus, strength, elongation at break, impact strength, transmittance after thermal annealing, and LOI values for neat PLLA and the biodegradable nanocomposites containing 20 wt% nanorods.

It is clear that the reaction between the end carboxylic acid group of PLLA with the epoxide groups on the surface of the boehmite nanorods is critically important to achieve the multifunctional performance of the final nanocomposites. We have made systematic investigations on the final PLLA chain bonded nanorods and the grafting ratio, as well as the calculated grafting density, is 41.7 wt % and 0.027 chains/nm^2^, respectively (Figure S3). Note that grafting ratio/density for the nanocomposites with various contents of AG nanorods is identical as all reactive epoxide groups in AG nanorods have been reacted with the terminal carboxyl groups of PLLA during processing. The therefore homogeneous dispersion and formation of nanorods networks in the PLLA matrix guaranteed the transparency, flame retardance, heat resistance, and the dimensional stability of the final nanocomposites. However, it is necessary to discuss the phenomena of the simultaneously increased tensile strength and impact strength of the nanocomposites by the nanorods. Simultaneously enhanced tensile strength and impact strength have seldom been achieved for the binary nanocomposites so far because most of the inorganic fillers increase Young’s modulus and tensile strength with sacrificing the ductility and toughness (Song and Wang, 2020; Yu et al., 2005; Zhang et al., 2019). The simultaneous increase in both tensile strength and impact strength has been pursued for a long time for polymer scientists. In this work, we attributed the drastically enhanced impact strength and ductility with nanorods to the occurrence of the forced high-elastic deformation on the stretching and/or impacting. PLLA chains are rigid and the neat PLLA is brittle at room temperature because *T*_*g*_ is higher than room temperature. The grafting of the PLLA chains onto the nanorods and the following dispersion of the nanorods in the PLLA matrix leads to a slightly decreasing in the *T*_*g*_ and a wider relaxation peak of PLLA in the matrix (Figure 5I). We consider that the decreased *T*_*g*_ can be attributed to the slightly increased free volume of PLLA because of the PLLA chain one end covalently grafting. As depicted in Figure 7, one end fixing definitely restrain the chain motions. However, the interfacial region should present increased free volume because of extended PLLA chains near the surface of the nanorods (Figure 7A). This is originated from the immiscibility between the hydrophobic PLLA chains and hydrophilic surface of nanorods. Such enlarged free volume of PLLA accounts for the decreased *T*_*g*_ of PLLA and the wide relaxation peak as well. More important, the changes in the PLLA molecular conformation and the free volume induce totally different tensile and impact behaviors on the deformations. The forced high-elastic deformation occurs for the nanorods dispersed nanocomposites (with a certain content of nanorods) on the tensile and/or impact deformation. PLLA chains were forced to move/orient along the external force (Figure 7B). Once the mobility of the molecular chains was induced by the external force, the large deformation or adsorption of massive impact energy occurs during the stretching or impact Figure 7C). We have successfully achieved the simultaneously enhanced Young’s modulus, tensile yielding strength, elongation at break, and impact strength for the biodegradable PLLA/nanorods composites.

Moreover, the perfect dispersion of nanorods leads to the significantly enhanced heat resistant, dimensional stability, improved flame retardance, and excellent transparency even after the full crystallization of the PLLA matrix. As shown in Figure 7D, one can find the dramatical enhancement in the physical properties (including mechanical, optical, and thermal properties) of the PLLA nanocomposites with 20 wt % nanorods as compared with those of the neat PLLA. Such multifunctional nanocomposites with a feasible fabrication strategy are expected to fulfill the requirements of bioplastics in the current situation. Moreover, the fabrication of such nanocomposites is feasible in industry and it should be easy to scale up. These results open a feasible strategy for the biodegradable PLLA materials not only for the packaging applications but also for the high-value engineering plastics. It brings a new possibility for high-performance biobased plastics.

### Limitations of the study

A limitation of this study is that it did not establish a direct mathematical correlation between decreased free volumes of polymer matrix and mechanical parameters of the bio-nanocomposites. Therefore, the function of rigid nanorods in mechanical strengthening should be further verified. Moreover, the idea should be further developed into a bio-nanocomposites system with higher aspect ratio of nanorods for verification and application in the sustainable packaging industry.

## STAR★Methods

### Key resources table

**Table:** 

REAGENT or RESOURCE	SOURCE	IDENTIFIER
**Chemicals, peptides, and recombinant proteins**		

Polylactic acid (PLLA)	Nature Works (USA)	Cat# 3001D-17
Aluminium isopropoxide	Aldrich	Cat# 229407
γ-(2,3-epoxypropoxy) propyltrimethoxysilane	Sinopharm Chemical Reagent	Cat# KH560
Acetic acid	Alfa	Cat# 010994.AP
Anhydrous ethanol	Sinopharm Chemical Reagent	Cat# 80059462
Petroleum ether	Sigma-Aldrich	Cat# 184519
Chloroform	Sinopharm Chemical Reagent	Cat# 10006818

### Resource availability

#### Lead contact

Further information and requests for resources and reagents should be directed to and will be fulfilled by the lead contact, Yongjin Li (yongjin-li@hznu.edu.cn).

#### Materials availability

This study did not generate new unique reagents.

### Method details

#### Synthesis of modified boehmite nanorods

Pure boehmite nanorods (AlOOH nanorods) were prepared by hydrothermal method from aluminum isopropoxide. At the end of the hydrothermal reaction, acetic acid and water were removed by rotary evaporation to obtain pure AlOOH. Next, modified boehmite nanorods (AG nanorods) were obtained by modifying the surface of boehmite nanorods with epoxy groups by silane coupling agent (GPS). The specific steps were as follows: AlOOH nanorods (5g) and GPS (10mL) were added into 250 mL anhydrous ethanol, and then ultra-sonicated for 5 min and 6 times. The reactants were transferred to a round-bottom flask and refluxed at 85°C for 20 h. After the reaction, the product was precipitated with petroleum ether and centrifuged at 5000 rpm for 5 min to remove the unreacted GPS, and finally modified boehmite nanorods were obtained. The modified AG nanorods were pre-dispersed in PLLA with chloroform to prevent the aggregation of nanorods. A typical procedure is as follows: AG nanorods (12 g) and PLLA (40 g) were simultaneously dispersed into 350 mL of chloroform and ultra-sonicated for 10 min. Then, the homogeneous solution was casted in PTFE container. The resultant mixture were dried in fuming cupboard at room temperature for 24 h and in vacuum at 65°C for 24 h for solvent evaporation.

#### Preparation of PLLA nanocomposites

PLLA were previously dried in a vacuum oven at 80°C for at least 12 h. Both PLLA and the PLLA/AG pre-mixer with a certain amount was simultaneously melt-blended in a batch mixer (Haake Polylab QC) at the mixing temperature of 190°C and the rotation speed of 50 rpm for 10 min to obtain the PLLA nanocomposites. In this work, the mass of PLLA is fixed at 50 g, and the concentration of nanorods is 0-30 wt%. The concentration of nanorods is regard to the sum of PLA and nanorods (Table S1). After melt blending, a part of samples were molded under a pressure of 10 MPa and at a hot pressing temperature of 200°C, and kept for 6 min. Finally, it was cooled to room temperature with ice-water, and two kinds of thin slices with thickness of 100 and 500 μm were obtained for characterization. In addition, splines for impact test were prepared using a micro-injection molding machine (Haake Minijet Pro) at a barrel temperature of 200°C and a mold temperature of 80°C.

#### Characterization of *PLLA nanocomposites*

*Fourier Transformed Infrared Resonance (FT-IR)*: FT-IR was performed by using Bruker VERTEX 70V. The samples were mixed with KBr and pressed into thin sheets, and then spectra were scanned from 4000 to 400 cm^−1^ with a resolution of 4 cm^−1^ for 64 times under vacuum at room temperature.

*Thermo-gravimetric analyses (TGA):* Thermo-gravimetric analysis (TGA) was performed by using TGA Q500 (TA Instruments, USA) in an atmosphere of nitrogen. Approximately 5 mg of samples were placed in a sample tray at a heating rate of 10°C/min ranging from 30 to 650°C. The mass ratio of grafted epoxy groups in AG nanorods can be calculated by the following Equation:

$$\varphi_{Epoxy}=\varphi_{AlOOH\;nanorods}-\varphi_{AG\;nanorods}$$

Here $\varphi_{Epoxy}$ referred to mass ratio of grafted epoxy groups, $\varphi_{AlOOH\;nanorods}$ and $\varphi_{AG\;nanorods}$ represented the inorganic content from AlOOH nanorods and AG nanorods. ($\varphi_{AlOOH\;nanorods}=81.6\%$ and $\varphi_{AG\;nanorods}=74.2\%$)

$$\varphi_{Epoxy}=81.6\%-74.2\%=7.4\%$$

The mass ratio of grafted PLLA chains on grafted nanorods can be calculated by following Equation:

$$\varphi_{PLLA}=\varphi_{AG\;nanorods}-\varphi_{grafted\;nanorods}$$

Here $\varphi_{PLLA}$ referred to mass ratio of grafted PLLA chains, $\;\varphi_{AG\;nanorods}$ and $\varphi_{grafted\;nanorods}$ represented the inorganic content from AG nanorods and grafted nanorods. ($\varphi_{AG\;nanorods}=74.2\%$ and $\varphi_{grafted\;nanorods}=32.5\%$)

$$\varphi_{PLLA}=74.2\%-32.5\%=41.7\%$$

The graft density of the grafted PLLA chains on the grafted nanorods can be further calculated as follows:

$${\text{σ}}_{PLLA}=\frac{f_{PLLA}N_{A}\rho_{nanorods}d}{4f_{nanorods}M_{n}}$$

Here $\sigma_{PLLA}$ referred to graft density of grafted PLLA chains; $f_{PLLA}$ and $f_{nanorods}$ represented the weight fraction of PLLA and boehmite nanorods of the grafted nanorods determined by TGA measurement; N_A_ is Avogadro’s number; $\rho_{nanorods}$ is the density of the boehmite nanorods; d is the average diameter of the boehmite nanorods; M_n_ is the number-average molecular of PLLA. ($f_{PLLA}$ =58.3% and $f_{PLLA}$ = 41.7%) ($\rho_{nanorods}$ is 3.43 g/cm^3^)

$${\text{σ}}_{PLLA}=\frac{f_{PLLA}N_{A}\rho_{nanorods}d}{4f_{nanorods}M_{n}}=0.027chain/nm^{2}$$

*Scanning Electron Microscopy (SEM):* The phase morphology was observed by scanning electron microscopy (SEM, Hitachi S-4800) at an accelerating voltage of 5.0 kV. All samples of the blends were immersed in liquid nitrogen for 10 min, quenched and then dried in a vacuum oven at 55°C for more than 2 h. The dried samples were pre-sprayed with gold for 15 s at room temperature under a vacuum environment.

*Transmission electron microscopy (TEM):* Transmission electron microscopy (TEM) was performed by Hitachi HT-7700 instrument at an accelerating voltage of 60 kV. All samples of the blends were ultramicrotomed to a thickness of 70-80 nm in liquid nitrogen at −120°C, and then drying in a vacuum oven at 55°C for more than half an hour in advance.

*Differential Scanning Calorimetry (DSC):* The crystallization and melting behaviors of the blends were characterized by DSC Q2000 (TA Instrument, USA) under a nitrogen atmosphere. All samples (about 5 mg) of the blends were placed in sample trays and heated from 30°C to 190°C at a heating rate of 10°C/min, then kept for 5 min to eliminate the thermal history, cooled to 30°C at a rate of 3°C/min, and finally increased to 200°C at a rate of 10°C/min, so as to analyze the crystallization and melting behavior of the sample. The crystallinity of PLLA matrix (X_c_) be evaluated by the following Equation:

$$X_{c}=\frac{\Delta H_{m}-\Delta H_{cc}}{\Delta H_{m}^{0}\times w_{f}}\times 100\%$$

Here $\Delta H_{m}$ referred to the enthalpy of melting of PLA; $w_{f}$ represented the weight fraction of the PLLA component; $\Delta H_{m}^{0}$ is the melting enthalpy of 100% crystalline polymer (93.7 J/g for PLA).

*Dynamic Mechanical Analysis (DMA):* Dynamic mechanical analysis (DMA) was performed using DMA Q800 (TA Instruments, USA) under a nitrogen atmosphere. All samples of blends were cut into a rectangle shape (14.0 × 6.20 × 0.50 mm) in advance., and heated from −40 to 250°C at a heating rate of 3°C/min at an amplitude of 5 μm and a frequency of 5 Hz.

*Mechanical Test:* Samples were prepared into dumbbell-shaped splines (20 mm length, 4 mm width, 2 mm thickness) by a micro-injection molding machine (Haake MiniJet Pro). Tensile testing was performed using an INSTRON Universal Material Testing System 5966 (Instron, USA) at a tensile rate of 10 mm/min at room temperature after placed for 12 h at room temperature to remove the stress concentration. The impact test was performed using an impact tester (SS-3700) and a pendulum energy of 4 J according to the GB/T 16420-1997 standard. Samples were prepared by a Haake Minijet Pro into standard splines with a length of 80mm, a width of 10mm and a thickness of 4 mm, and then placed for 24 h. Finally each sample was tested at least five times to obtain an average result with standard deviation.

*Light transmittance and Haze Test:* The light transmittance of the blend was characterized by light transmittance/haze tester (Shen Guang Instruments, WGT-S). The blends were tested for light transmittance and haze by selecting 5 distinct areas in a 100 μm sheet and the final results were the average of the data.

*Refractive Index Test:* The refractive index of the films with a thickness of 100 μm was measured by an Abbe Refractometer (NAR-1T SOLID) under a sodium light source (wave number 589.3 nm). Each group of samples was measured 5 times, and the final results were taken as the average value.

*Wide-Angle X-Ray Diffraction:* The crystal structure of the blends was characterized by Bruker D8 (Bruker, Germany) with the radioactive source of CuKα (λ = 0.154 nm). Measured by a wide-angle X-ray diffractometer at a scanning rate of 2 º/min within a diffraction angle (2θ) of 5-50°.

*Polarized Optical Microscopy (POM):* The crystal morphology of the materials was observed by a phase difference polarized optical microscope (POM, Olympus BX-51) and the temperature was controlled using a Linkham LTS 350 hot stage. Samples with a thickness of 100 μm were placed on clean glass sheets, melted and pressed to the thinnest, and then observed during isothermal crystallization at 130°C.

*Flame retardancy measurements:* Limiting oxygen index (LOI) measurements were tested according to ASTM D2863 standard. The value of the LOI is carried out in the HC-2C (Jiang Ning analytical instrument, China). All samples have the size of 80 mm (length)×10 mm (width)×4 mm (thickness).

## Data Availability

• Data reported in this paper will be shared by the lead contact upon request. • This paper does not report original code • Any additional information required to reanalyze the data reported in this paper is available from the lead contact upon request.

## Supplemental information

Document S1. Figures S1–S11, Tables S1–S11, and Equation S1

## References

[bib1] Altman R. (2021). The myth of historical bio-based plastics. Science.

[bib2] Amirian M., Nabipour Chakoli A., Sui J.H., Cai W. (2013). Thermo-mechanical properties of MWCNT-g-poly(l-lactide)/poly(l-lactide) nanocomposites. Polym. Bull..

[bib3] Borba N.Z., Körbelin J., Fiedler B., Dos Santos J.F., Amancio-Filho S.T. (2020). Low-velocity impact response of friction riveted joints for aircraft application. Mater. Des..

[bib4] Boutry C.M., Kaizawa Y., Schroeder B.C., Chortos A., Legrand A., Wang Z., Chang J., Fox P., Bao Z. (2018). A stretchable and biodegradable strain and pressure sensor for orthopaedic application. Nat. Electron..

[bib5] Brahney J., Hallerud M., Heim E., Hahnenberger M., Sukumaran S. (2020). Plastic rain in protected areas of the United States. Science.

[bib6] Chen J., Rong C., Lin T., Chen Y., Wu J., You J., Wang H., Li Y. (2021). Stable co-continuous PLA/PBAT blends compatibilized by interfacial stereocomplex crystallites: toward full biodegradable polymer blends with simultaneously enhanced mechanical properties and crystallization rates. Macromolecules.

[bib7] Das K., Sinha Ray S., Chapple S., Wesley-Smith J. (2013). Mechanical, thermal, and fire properties of biodegradable polylactide/boehmite alumina composites. Ind. Eng. Chem. Res..

[bib8] Ding H., Yang W., Yu W., Liu T., Wang H., Xu P., Lin L., Ma P. (2021). High hydrophobic poly (lactic acid) foams impregnating one-step Si–F modified lignin nanoparticles for oil/organic solvents absorption. Compos. Commun..

[bib9] Dong W., Jiang F., Zhao L., You J., Cao X., Li Y. (2012). PLLA microalloys versus PLLA nanoalloys: preparation, morphologies, and properties. ACS Appl. Mater. Interfaces.

[bib10] Elsawy M.A., Saad G.R., Sayed A.M. (2016). Mechanical, thermal, and dielectric properties of poly(lactic acid)/chitosan nanocomposites. Polym. Eng. Sci..

[bib11] Lizundia E., Fortunati E., Dominici F., Vilas J.L., León L.M., Armentano I., Torre L., Kenny J.M. (2016). PLLA-grafted cellulose nanocrystals: role of the CNC content and grafting on the PLA bionanocomposite film properties. Carbohydr. Polym..

[bib12] Fallahi H., Azizi H., Ghasemi I., Karrabi M. (2017). Preparation and properties of electrically conductive, flexible and transparent silver nanowire/poly (lactic acid) nanocomposites. Org. Electron..

[bib13] Fu Z., Wang H., Zhao X., Li X., Gu X., Li Y. (2019). Flame-retarding nanoparticles as the compatibilizers for immiscible polymer blends: simultaneously enhanced mechanical performance and flame retardancy. J. Mater. Chem..

[bib14] Gao Q., Zhao X., Wang C., Wang L., Ma Z. (2018). Multi-objective crashworthiness optimization for an auxetic cylindrical structure under axial impact loading. Mat. Des..

[bib15] Geyer R., Jambeck J.R., Law K.L. (2017). Production, use, and fate of all plastics ever made. Sci. Adv..

[bib16] Gu L., Qiu J., Qiu C., Yao Y., Sakai E., Yang L. (2019). Mechanical properties and degrading behaviors of aluminum hypophosphite-poly (Lactic Acid)(PLA) nanocomposites. Polym.-Plast. Tech. Mat..

[bib17] Hamad K., Kaseem M., Ayyoob M., Joo J., Deri F. (2018). Polylactic acid blends: the future of green, light and tough. Prog. Polym. Sci..

[bib18] Hu G., Hoppe S., Feng L., Fonteix C. (2007). Nano-scale phenomena and applications in polymer processing. Chem. Eng. Sci..

[bib19] Hu G.H., Triouleyre S., Lambla M. (1997). Kinetic behaviour of chemical reactions in homogeneous and heterogeneous polymer melts. Polymer.

[bib20] Hu L., Fu Z., Gu X., Wang H., Li Y. (2021). Strengthened interface as flame retarding belt: compatibilized PLLA/PP blends by reactive boehmite nanorods. Polymer.

[bib21] Huang L., Tan J., Li W., Zhou L., Liu Z., Luo B., Lu L., Zhou C. (2018). Functional polyhedral oligomeric silsesquioxane reinforced poly(lactic acid) nanocomposites for biomedical applications. J. Mech. Behav. Biomed. Mater..

[bib22] Jia J., Yang J., Zhao Y., Liang H., Chen M. (2016). The crystallization behaviors and mechanical properties of poly(L-lactic acid)/magnesium oxide nanoparticle composites. RSC Adv..

[bib23] Li C., Liu H., Luo B., Wen W., He L., Liu M., Zhou C. (2016). Nanocomposites of poly(l-lactide) and surface-modified chitin whiskers with improved mechanical properties and cytocompatibility. Eur. Polym. J..

[bib24] Li H., Cao Z., Wu D., Tao G., Zhong W., Zhu H., Qiu P., Liu C. (2016). Crystallisation, mechanical properties and rheological behaviour of PLA composites reinforced by surface modified microcrystalline cellulose. Plast., Rubber Compos..

[bib25] Liang H., Zhao Y., Yang J., Li X., Yang X., Sasikumar Y., Zhou Z., Chen M. (2019). Fabrication, Fabrication, crystalline behavior, mechanical property and in-vivo degradation of poly(l–lactide) (PLLA)–magnesium oxide whiskers (MgO) nano composites prepared by in-situ polymerization. Polymers.

[bib26] Jiang B., Chen C., Liang Z., He S., Kuang Y., Song J., Mi R., Chen G., Jiao M., Hu L. (2020). Lignin as a wood-inspired binder enabled strong, water stable, and biodegradable paper for plastic replacement. Adv. Funct. Mater..

[bib27] Jin F.L., Hu R.R., Park S.J. (2019). Improvement of thermal behaviors of biodegradable poly (lactic acid) polymer: a review. Composites Part B.

[bib28] Jordan A.M., Kim K., Soetrisno D., Hannah J., Bates F.S., Jaffer S.A., Lhost O., Macosko C.W. (2018). Role of crystallization on polyolefin interfaces: an improved outlook for polyolefin blends. Macromolecules.

[bib29] Kakadellis S., Rosetto G. (2021). Achieving a circular bioeconomy for plastics. Science.

[bib30] Law K.L., Starr N., Siegler T.R., Jambeck J.R., Mallos N.J., Leonard G.H. (2020). The United States’ contribution of plastic waste to land and ocean. Sci. Adv..

[bib31] Li C., Adamcik J., Mezzenga R. (2012). Biodegradable nanocomposites of amyloid fibrils and graphene with shape-memory and enzyme-sensing properties. Nat. Nanotechnol..

[bib32] Li R., Zhao X., Coates P., Caton-Rose F., Ye L. (2021). Highly reinforced poly (lactic acid) foam fabricated by formation of a heat-resistant oriented stereocomplex crystalline structure. ACS Sustain. Chem. Eng..

[bib33] Li X., Fu Z., Gu X., Liu H., Wang H., Li Y. (2020). Interfacially located nanoparticles: barren nanorods versus polymer grafted nanorods. Composites Part B.

[bib34] Liu B., Li F., Niu P., Li H. (2021). Tough adhesion of freezing-and drying-tolerant transparent nanocomposite organohydrogels. ACS Appl. Mater. Interfaces.

[bib35] MacLeod M., Arp H.P.H., Tekman M.B., Jahnke A. (2021). The global threat from plastic pollution. Science.

[bib36] Mohanty A.K., Vivekanandhan S., Pin J.M., Misra M. (2018). Composites from renewable and sustainable resources: challenges and innovations. Science.

[bib37] Mu B., Yang Y. (2022). Complete separation of colorants from polymeric materials for cost-effective recycling of waste textiles. Chem. Eng. J..

[bib38] Muller J., González-Martínez C., Chiralt A. (2017). Combination of poly(Lactic) acid and starch for biodegradable food packaging. Materials.

[bib39] Pernot H., Baumert M., Court F., Leibler L. (2002). Design and properties of co-continuous nanostructured polymers by reactive blending. Nat. Mater..

[bib40] Rochman C.M., Cook A.M., Koelmans A.A. (2016). Plastic debris and policy: using current scientific understanding to invoke positive change. Environ. Toxicol. Chem..

[bib41] Rong C., Chen Y., Chen C., Hu L., Wang H., Li Y. (2022). Toward simultaneous compatibilization and nucleation of fully biodegradabe nanocomposites: effect of nanorod-assisted interfacial stereocomplex crystals in immiscible polymer blends. Composites Part B.

[bib42] Roy Goswami S., Sudhakaran Nair S., Wang S., Yan N. (2021). Recent progress on starch maleate/polylactic acid blends for compostable food packaging applications. ACS Sustainable Chem. Eng..

[bib43] Sato T., Dunderdale G.J., Hozumi A. (2020). Large-scale formation of fluorosurfactant-doped transparent nanocomposite films showing durable antifogging, oil-repellent, and self-healing properties. Langmuir.

[bib44] Sharma V., Chowdhury S., Bose S., Basu B. (2022). Polydopamine codoped BaTiO3-functionalized polyvinylidene fluoride coating as a piezo-biomaterial platform for an enhanced cellular response and bioactivity. ACS Biomater. Sci. Eng..

[bib45] Shi C., Zhou A., Fang D., Lu T., Wang J., Song Y., Lyu L., Wu W., Huang C., Li W. (2022). Oregano essential oil/β-cyclodextrin inclusion compound polylactic acid/polycaprolactone electrospun nanofibers for active food packaging. Chem. Eng. J..

[bib46] Song P., Wang H. (2020). High-performance polymeric materials through hydrogen-bond cross-linking. Adv. Mater..

[bib47] Toledo J.M., Aznar M.P., Sancho J.A. (2011). Catalytic air gasification of plastic waste (polypropylene) in a fluidized bed. Part II: effects of some operating variables on the quality of the raw gas produced using olivine as the in-bed material. Ind. Eng. Chem. Res..

[bib48] Vieira O., Ribeiro R.S., Diaz de Tuesta J.L., Gomes H.T., Silva A.M. (2022). A systematic literature review on the conversion of plastic wastes into valuable 2D graphene-based materials. Chem. Eng. J..

[bib49] Wang C.C., Wei S.C., Luo S.C. (2022). Recent advances and biomedical applications of peptide-integrated conducting polymers. ACS Appl. Bio Mater..

[bib50] Wang H., Zhao X., You J., Li Y. (2020). Porous nanocomposites with monolayer nano-SiO_2_ coated skeleton from interfacial nanoparticle-anchored cocontinuous polymer blends. ACS Appl. Polym. Mater..

[bib51] Wang L., Ma Z., Zhang Y., Chen L., Cao D., Gu J. (2021). Polymerbased EMI shielding composites with 3D conductive networks: a mini-review. SusMat.

[bib52] Wang L.N., Guo Wang P.Y., Wei J.C. (2017). Graphene oxide-graft-poly(l-lactide)/poly(l-lactide) nanocomposites: mechanical and thermal properties. Polymers.

[bib53] Weckhuysen B.M. (2020). Creating value from plastic waste. Science.

[bib54] Wen W., Luo B., Qin X., Li C., Liu M., Ding S., Zhou C. (2015). Strengthening and toughening of poly(L-lactide) composites by surface modified MgO whiskers. Appl. Surf. Sci..

[bib55] Wu F., Misra M., Mohanty A.K. (2021). Challenges and new opportunities on barrier performance of biodegradable polymers for sustainable packaging. Prog. Polym. Sci..

[bib56] Xia Q., Chen C., Yao Y., Li J., He S., Zhou Y., Li T., Pan X., Yao Y., Hu L. (2021). A strong, biodegradable and recyclable lignocellulosic bioplastic. Nat. Sustain..

[bib57] Ye L., Ye C., Xie K., Shi X., You J., Li Y. (2015). Morphologies and crystallization behaviors in melt-miscible crystalline/crystalline blends with close melting temperatures but different crystallization kinetics PLLA microalloys versus PLLA nanoalloys: preparation, morphologies, and properties. Macromolecules.

[bib58] Yu Z.Z., Hu G.H., Varlet J., Dasari A., Mai Y.W. (2005). Water-assisted melt compounding of nylon-6/pristine montmorillonite nanocomposites. J. Polym. Sci., Part B: Polym. Phys..

[bib59] Zaidy S.S.H., Vacchi F.I., Umbuzeiro G.A., Freeman H.S. (2019). Approach to waterless dyeing of textile substrates—use of atmospheric plasma. Ind. Eng. Chem. Res..

[bib60] Zhang Q., Mochalin V., Neitzel I., Hazeli K., Niu J., Kontsos A., Zhou J.G., Zhou J., Lelkes P.I., Lelkes P., Gogotsi Y. (2012). Mechanical properties and biomineralization of multifunctional nanodiamond-PLLA composites for bone tissue engineering. Biomaterials.

[bib61] Zhang X., Liu W., Yang D., Qiu X. (2019). Biomimetic supertough and strong biodegradable polymeric materials with improved thermal properties and excellent UV-blocking performance. Adv. Funct. Mater..

[bib62] Zhao X., Cornish K., Vodovotz Y. (2020). Narrowing the gap for bioplastic use in food packaging: an update. Environ. Sci. Technol..

[bib63] Zhao X., Korey M., Li K., Copenhaver K., Tekinalp H., Celik S., Kalaitzidou K., Ruan R., Ragauskas A.J., Ozcan S. (2022). Plastic waste upcycling toward a circular economy. Chem. Eng. J..

[bib64] Zhu W., Huang J., Lu W., Sun Q., Peng L., Fen W., Li H., Ou Y., Liu H., Wang D., Zeng Y. (2014). Performance test of Nano-HA/PLLA composites for interface fixation. Artifi. Cell. Nanomed. B..

[bib65] Zhu Y., Romain C., Williams C.K. (2016). Sustainable polymers from renewable resources. Nature.

[bib66] Zou Z., Luo C., Luo B., Wen W., Liu M., Zhou C. (2016). Synergistic reinforcing and toughening of poly(L-lactide) composites with surface-modified MgO and chitin whiskers. Compos. Sci. Technol..

