# Supplementary material for: Enhancement of strength and toughness of bio-nanocomposites with good transparency and heat resistance by reactive processing
Source: iScience. 2022 Jun 8;25(7):104560. doi: 10.1016/j.isci.2022.104560 (PMC9234255; doi:10.1016/j.isci.2022.104560)
Supplement: Document S1. Figures S1–S11, Tables S1–S11, and Equation S1 [file mmc1.pdf]

iScience, Volume *25*

Supplemental information

Enhancement of strength and toughness of bio-nanocomposites with good transparency and heat resistance by reactive processing

Hengti Wang, Chenyan Rong, Jichun You, and Yongjin Li

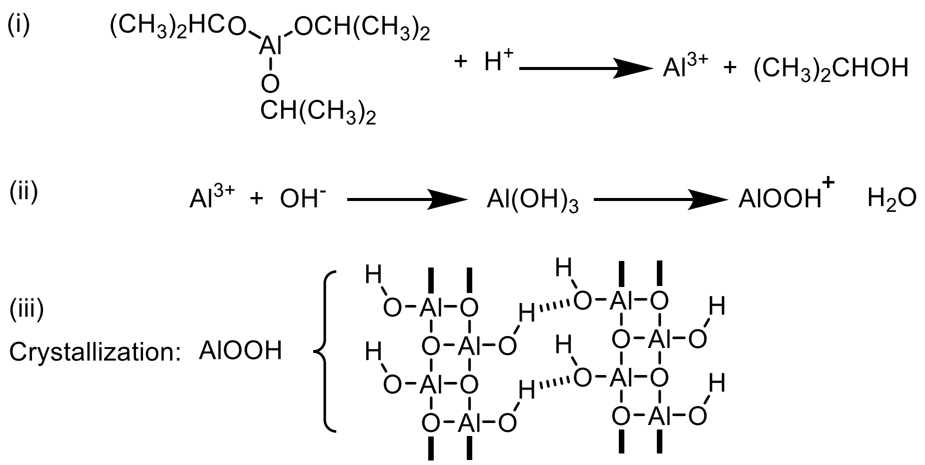

Equation S1. Synthesis of AlOOH nanorods through hydrotherm treatment

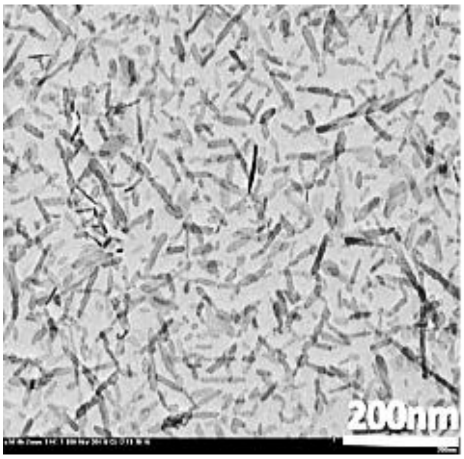

Figure S1. TEM image of AlOOH nanorods

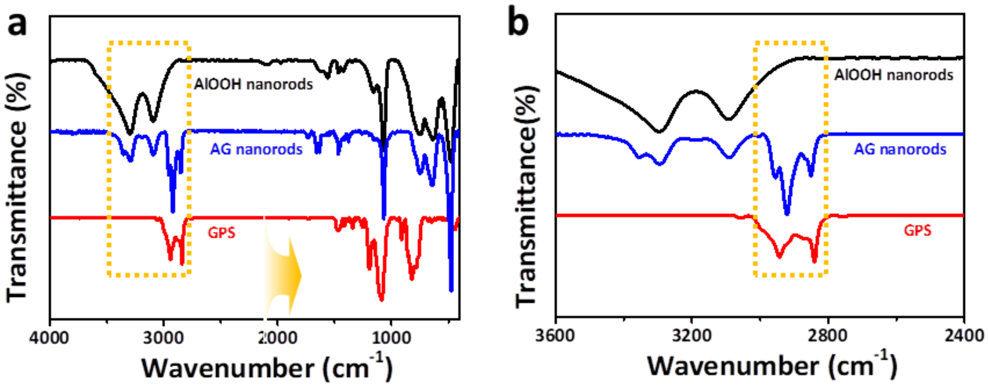

Figure S2. FT-IR spectra of pristine AlOOH nanorods, modified AG nanorods and the silane coupling agent GPS in the wavenumber region of **a** 4000-400 cm^{-1}, and **b** 3600-2400 cm^{-1}

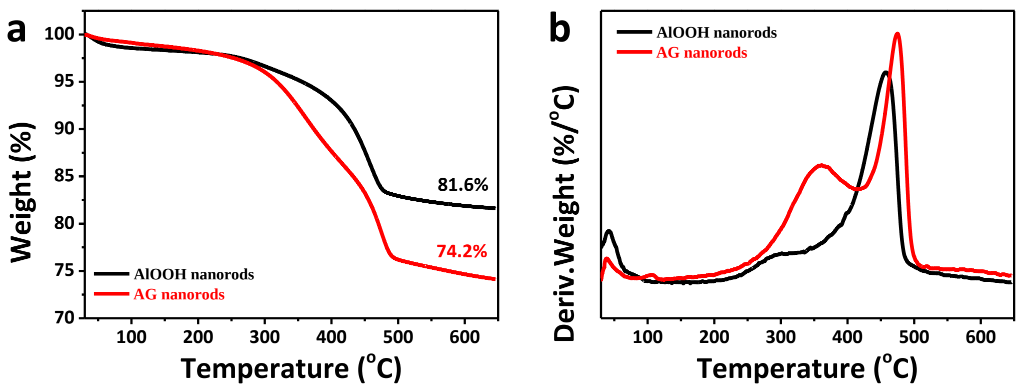

Figure S3. **a** TGA and **b** DTG curves of pristine AlOOH nanorods and modified AG nanorods

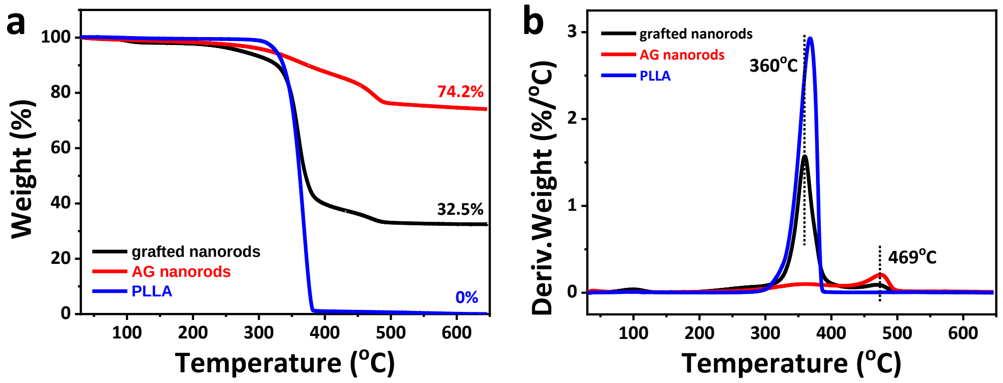

Figure S4. **a** TGA and **b** DTG curves of modified AG nanorods, grafted nanorods and PLLA

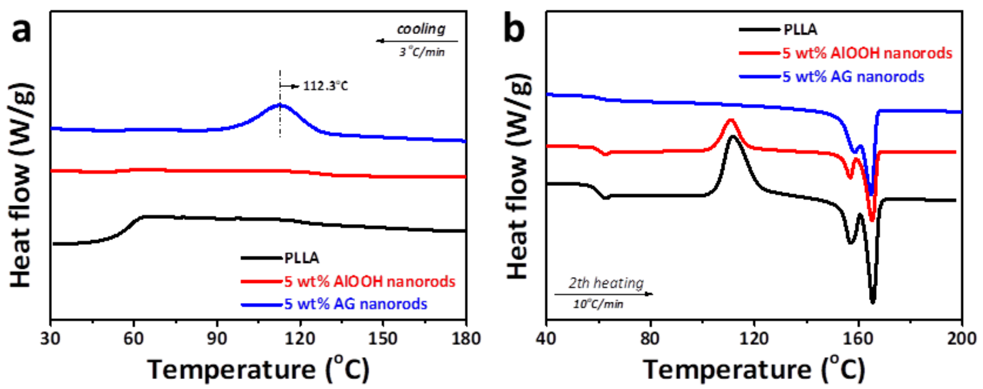

Figure S5. a DSC cooling curves, and **b** second heating curves of PLLA and PLLA nanocomposites incorporated with 5 wt% AlOOH and 5 wt% AG nanorods

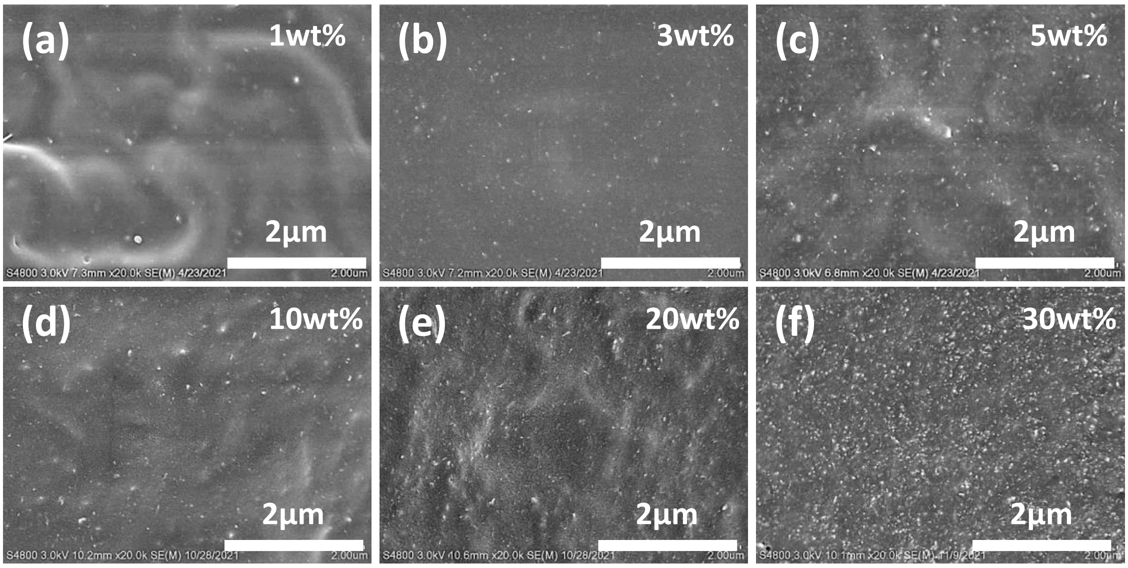

Figure S6. SEM images of PLLA nanocomposites with **a** 1 wt%, **b** 3 wt%, **c** 5 wt%, **d** 10 wt%, **e** 20 wt% and **f** 30 wt% nanorods

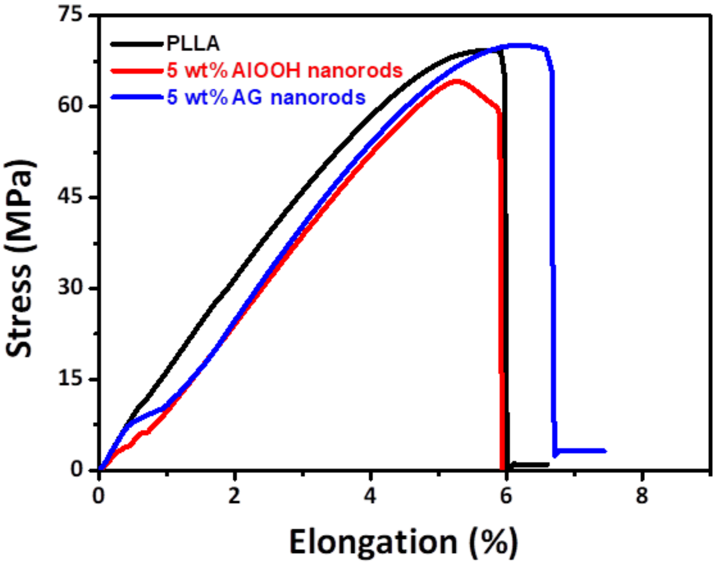

Figure S7. Typical stress−strain curves of pristine PLLA and the PLLA nanocomposites incorporated with 5 wt% AlOOH or 5 wt% AG nanorods

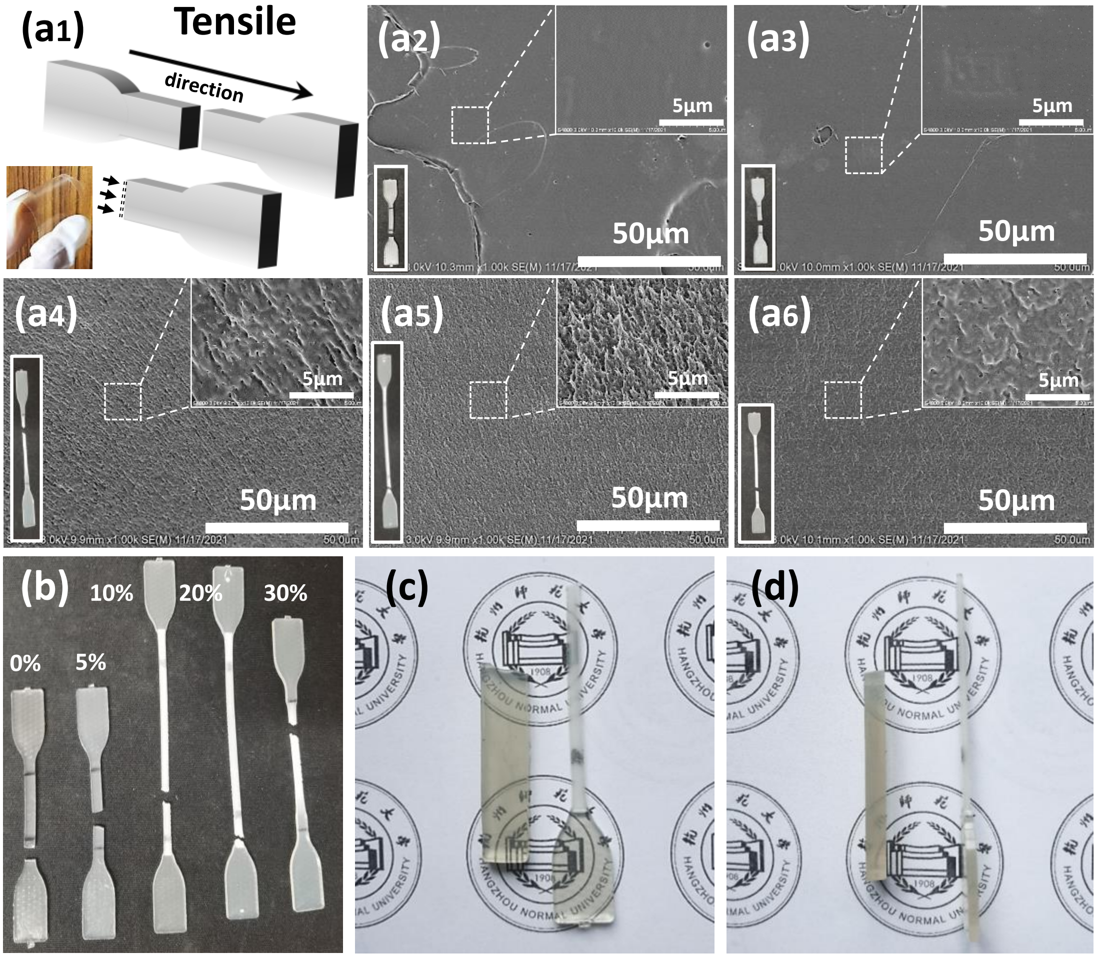

Figure S8. a1 SEM images of fractured sections of PLLA nanocomposites with **a2** 0 wt%, **a3** 5 wt%, **a4** 10 wt%, **a5** 20 wt%, and **a6** 30 wt% nanorods,and **b-d** Photographs of the injection samples after tensile and impact experiments.

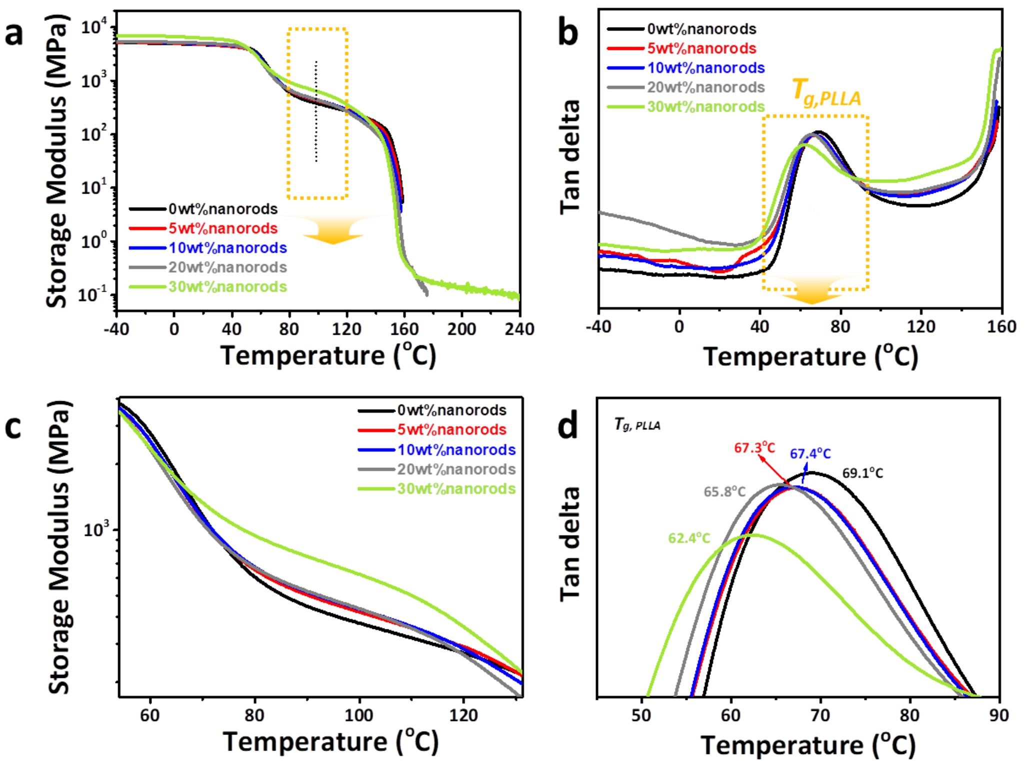

Figure S9. DMA curves of the PLLA nanocomposites with various content of nanorods after annealing: **a**. **c** Storage modulus and **b**. **d**. Tan delta in dependence of temperature

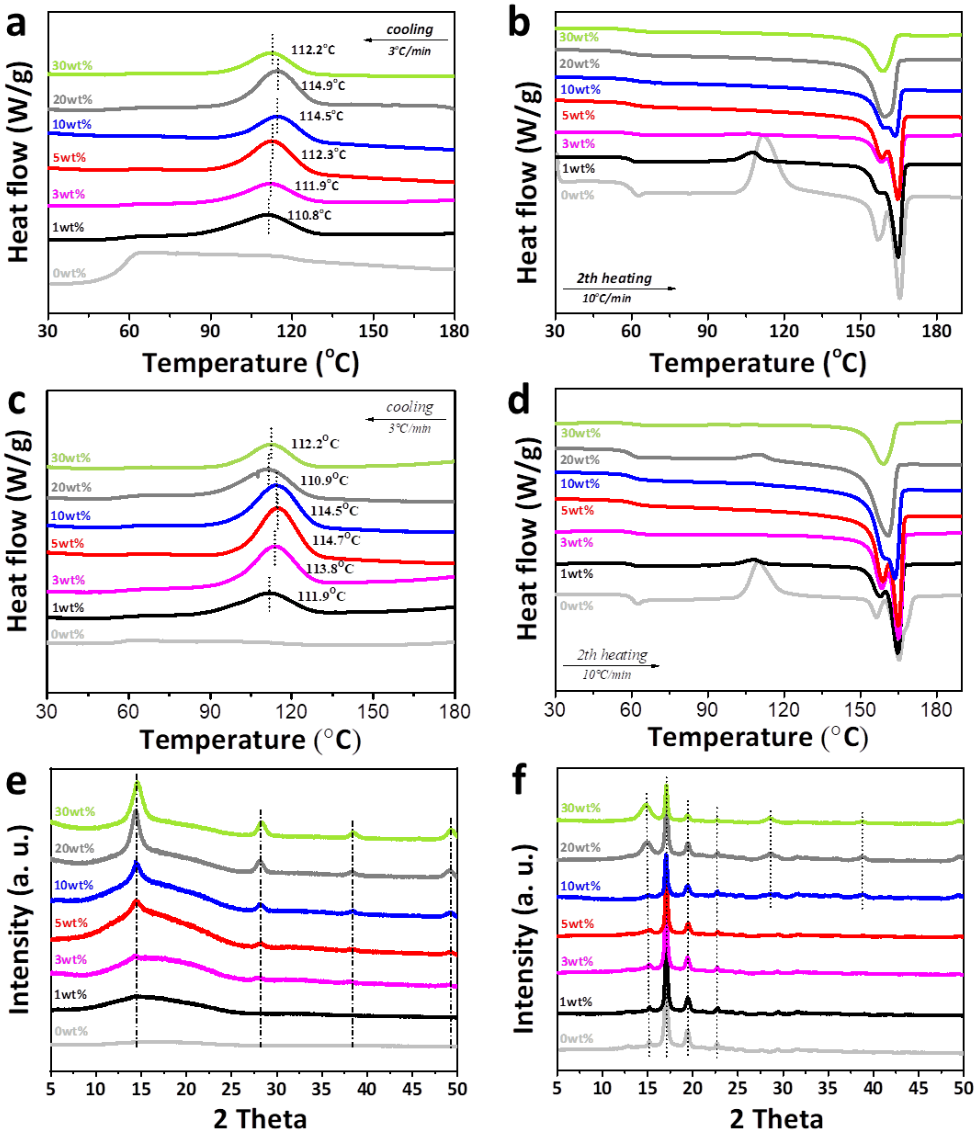

Figure S10. a. c DSC cooling curves, **b. d** second heating curves of PLLA nanocomposites with different content of nanorods **a. c** before and **c. d** after annealing, and XRD curves of PLLA nanocomposites with different content of nanorods **e** before and **f** after annealing

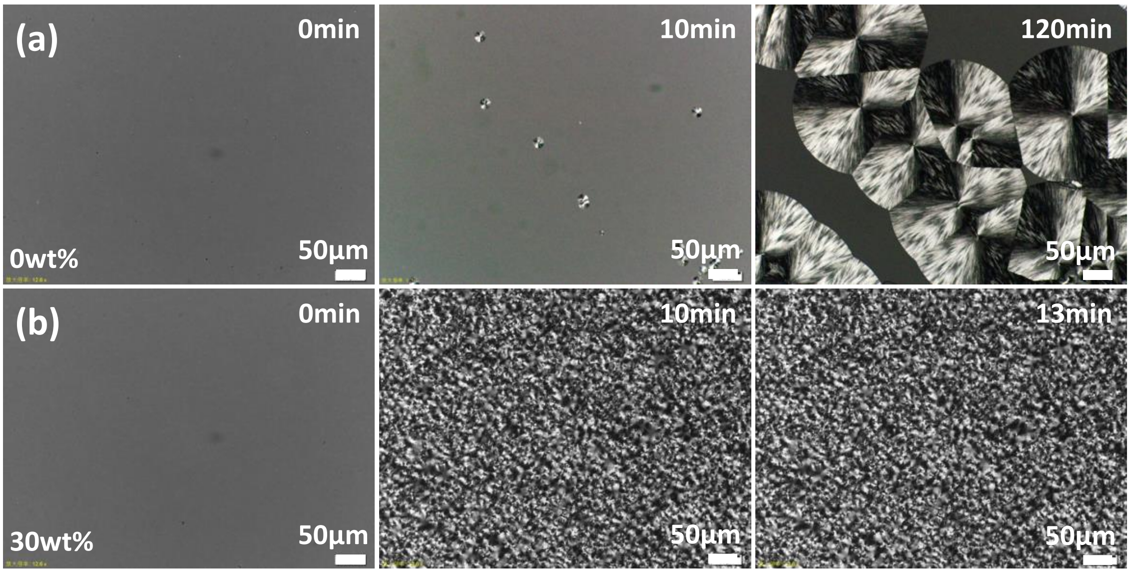

Figure S11. POM images of PLLA nanocomposites isothermal crystallized at 130 °C: **a** Pristine PLLA for 0, 10, 120 min; **b** Nanocomposites containing 30 wt% AG nanorods for 0, 10, 13 min

Table S1. Composition and nomenclature of PLLA nanocomposites with different content of nanorods

Code	Type of nanorods	Concentration of nanorods (wt%) [a]
0%nanorods	AG nanorods	0
1%nanorods	AG nanorods	1
3%nanorods	AG nanorods	3
5%nanorods	AG nanorods	5
10%nanorods	AG nanorods	10
20%nanorods	AG nanorods	20
30%nanorods	AG nanorods	30
5%AlOOH nanorods	AlOOH nanorods	5

[a] The concentration of nanorods is calculated by the following equation: $C_{nanorods} = \frac{w_{nanorods}}{w_{nanorods}+w_{PLLA}}$

Table S2. Mechanical parameters for PLLA and PLLA nanocomposites added with 5 wt% AlOOH and 5 wt% AG nanorods

**Sample**	**Tensile modulus(MPa)**	**Elongation at break(%)**	**Tensile strength at break(MPa)**
PLLA	1509.7±17.8	5.6±0.4	67.0±2.9
5%AlOOH nanorods	1538.6±58.5	4.3±0.5	53.0±4.8
5%AG nanorods	1546.7±56.7	6.3±0.3	69.5±0.6

Table S3. Mechanical parameters for PLLA nanocomposites with different content of nanorods

sample	Tensile modulus (MPa)	Tensile strength at yielding (MPa)	Tensile strength at break (MPa)	Elongation at break (%)
0%nanorods	1509.7±17.8	67.0±0.2	63.3±2.9	5.9±0.4
5%nanorods	1546.7±56.7	69.5±0.3	66.0±0.6	6.5±0.3
10%nanorods	1861.9±28.2	69.8±0.4	42.5±0.6	271.2±10.9
20%nanorods	1941.8±6.5	72.0±1.1	48.5±4.2	240.9±48.9
30%nanorods	2285.3±45.5	75.1±1.4	44.3±1.7	108.8±5.2

Table S4. Variation of mechanical parameters from pristine PLLA to PLLA-based nanocomposites and the related increasing rate

Ref.	Composition	transparency	Elongation at break	Tensile strength	Tensile modulus	Impact strength/energy
1	PLLA/CNC-g-PLLA	From 93.57% to 95.56%	From 5.6% to 9.5%（↑70%）	From 41 MPa to 43.5 MPa（↑6.1%）	From 1980 MPa to 2430 MPa（↑22.7%）	
2	Nano-HA/PLLA			From 55.6 MPa to38.6 MPa ↓		
3	PLLA-MgO		From 3.06% to 53%（↑1630%）	From 10 MPa to 16.8 MPa（↑68%）	From 611 MPa to 1640 MPa（↑168%）	
4	PLLA/POSS-(PLLA)		From 9.38% to 10.4%（↑10.9%）	From 33.7 MPa to 42.7 MPa（↑27%）	From 1096 MPa to 1721MPa（↑57%）	From 206.1MJ/m^2 to 263.3 MJ/m^2（↑27.8%）
5	g-CHW/PLLA		From 6.03% to 13.9%（↑130.1%）	From 18.7 MPa to 30.5 MPa（↑63.1%）	From 0.6 GPa to 1.4 GPa（↑133.3%）	From 64.77 J/mm to 333.7 J/mm（↑415%）
6	PLLA / m-MgO-NP		From 5% to 46%（↑820%）	From 28 MPa to 45 MPa（↑60.7%）	From 1330 MPa to 2300 MPa（↑72.9%）	
7	ND-ODA/PLLA		From 5% to 19%（↑280%）	From 53 MPa to 54 MPa（↑1.9%）	From 3.1 GPa to 2.7 GPa ↓	From 119 J to 827 J（↑316%）
8	PLA/CsNP		From 3.3% to 7.7%（↑133%）	From 74.1 MPa to 53.1 MPa ↓	From 3.41 GPa to 3.69 GPa（↑8.2%）	From 13.1KJ/m^2 to 17.1 KJ/m^2（↑30.5%）
9	PLLA/g-CHN-Ws		From 7.3% to 10.1%（↑38%）	From 63.4 MPa to 72.7 MPa（↑14.7%）	From 1126 MPa to 1243 MPa（↑10.4%）	From 23.1 J/m to 30.4 J/m（↑31.6%）
10	AgNW/PLA	From 96.5%to 89.2%↓		From 33 MPa to 39.8 MPa（↑20.6%）	From 1535 MPa to 1589MPa（↑3.5%）	
11	GO-g-PLLA/PLLA			From 51 MPa to 60 MPa（↑37.8%）		
12	PLA/K-MCC			From 62.9 MPa to 71 MPa（↑12.9%）	From 3.72 GPa to 4.65 GPa（↑25%）	
13	PLLA/AHP		From 5.9% to 5.9%	From 68.8 ± 2.1 MPa to 56.3 ± 1.6 MPa ↓	From 2500 MPa to 11700 MPa（↑360%）	From 2.35 ± 0.17 kJ·m^{-2} to 2.9 ± 0.19 kJ·m^{-2}
14	g-MgO/PLLA		From 56.52% to 74.11%（↑31.1%）	From 38 MPa to 68MPa（↑79%）	From 1.59 GPa to 2.10 GPa（↑32%）	From 2000 J/mm to 4900 J/mm（↑145%）
15	MWCNT-g-PLLAs/PLLA		From 157% to 285%（↑81%）	From 32.3 MPa to 38.3 MPa (↑47 %)	From 4.1 MPa to 5.1 MPa（↑24%）	
	This work	From 93.2% to 91.2%↓	From 5.6% to 261.2%（↑4564%）	From 67 MPa to 72.4 MPa (↑8 %)	From 1509.7 MPa to 2285.3 MPa (↑51.4 %)	From 2.2 KJ/m^2 to 35.8 KJ/m^2（↑1527%）

Table S5. Light transmittance and haze parameters for PLLA nanocomposites before annealing

Sample	Transmittance (%)	Haze (%)
0%nanorods	93.2	2.1
1%nanorods	93.1	2.2
3%nanorods	93.0	2.3
5%nanorods	92.8	2.7
10%nanorods	92.5	2.8
20%nanorods	92.1	2.9
30%nanorods	91.2	3.1

Table S6. Light transmittance and haze parameters for PLLA nanocomposites after annealing

**Sample**	**Transmittance (%)**	**Haze (%)**
0%nanorods	84.2	87.6
1%nanorods	90.7	53.8
3%nanorods	91.4	47.5
5%nanorods	91.8	38.7
10%nanorods	91.8	32.3
20%nanorods	91.6	30.6
30%nanorods	91.1	27.2

Table S7. Refractive index parameters for PLLA nanocomposites after annealing

Sample	Refractive index
AlOOH	1.631±0.000631
0%nanorods	1.455±0.000565
3%nanorods	1.456±0.00065
5%nanorods	1.460±0.000463
10%nanorods	1.464±0.000625
20%nanorods	1.466±0.000225
30%nanorods	1.474±0.000125

Table S8. Thermal Parameters of PLLA nanocomposites with different content of nanorods after annealing obtained from DMA results[a]

Sample	Storage Modulus(MPa) (25℃)	Storage Modulus(MPa) (83℃)	Storage Modulus(MPa) (120℃)	$T_{g,PLLA}$(℃)
0%nanorods	4693	540.4	271	69.1
5%nanorods	4710	596.3	290	67.4
10%nanorods	4862	613.6	283	67.3
20%nanorods	4956	613.2	266	65.8
30%nanorods	6143	873.6	358	62.4

[a] Data from DMA.

Table S9. Thermal Parameters of PLLA nanocomposites with different content of nanorods obtained from DMA results[a]

**Sample**	**Storage Modulus(MPa) (25℃)**	**Storage Modulus(MPa) (83℃)**	**Storage Modulus(MPa) (120℃)**	**$T_{g,PLLA}$(℃)**
0%nanorods	3346	1.7	82	63.9
5%nanorods	4200	3.8	155	63.9
10%nanorods	4701	5.8	207	63.5
20%nanorods	5175	6.4	135	62.6
30%nanorods	5296	8.8	228	61.5

[a] Data from DMA.

Table S10. Thermal Parameters of PLLA nanocomposites with different content of nanorods after annealing from DSC results[a]

Sample	T_c(℃)	T_m(℃)		ΔH_m(J/g)	X_c(%)
**0%nanorods**	/	156.4	165.1	3.54	3.8
**1%nanorods**	111.9	158.0	164.7	35.95	38.9
**3%nanorods**	113.8	158.5	165.1	37.90	41.8
**5%nanorods**	114.7	158.8	165.0	36.60	41.1
**10%nanorods**	114.5	160.1	163.6	30.53	36.0
**20%nanorods**	110.9	161.1		24.76	31.8
**30%nanorods**	112.2	159.3		21.81	30.4

[a] Data from DSC measurements.

Table S11. Thermal Parameters of PLLA nanocomposites with different content of nanorods from DSC results[a]

Sample	T_c(℃)	T_m(℃)		ΔH_m(J/g)	X_c(%)
**0%nanorods**	/	157.0	165.6	0.64	0.7
**1%nanorods**	110.8	158.4	164.9	31.28	33.8
**3%nanorods**	111.9	158.5	164.9	33.95	37.4
**5%nanorods**	112.3	158.4	164.7	34.88	39.2
**10%nanorods**	114.5	159.7	163.6	32.62	38.4
**20%nanorods**	114.9	159.5		28.72	36.9
**30%nanorods**	112.2	158.7		23.27	32.4

[a] Data from DSC measurements.
